# Bioactive Compounds from Guava Leaves (*Psidium guajava* L.): Characterization, Biological Activity, Synergistic Effects, and Technological Applications

**DOI:** 10.3390/molecules30061278

**Published:** 2025-03-12

**Authors:** Hoang Duy Huynh, Parushi Nargotra, Hui-Min David Wang, Chwen-Jen Shieh, Yung-Chuan Liu, Chia-Hung Kuo

**Affiliations:** 1 Department of Seafood Science, National Kaohsiung University of Science and Technology, Kaohsiung 81157, Taiwan; i113199116@nkust.edu.tw (H.D.H.); parushi11nargotra@gmail.com (P.N.); 2 Institute of Aquatic Science and Technology, National Kaohsiung University of Science and Technology, Kaohsiung 81157, Taiwan; 3 Graduate Institute of Biomedical Engineering, National Chung Hsing University, Taichung 402, Taiwan; davidw@dragon.nchu.edu.tw; 4 Biotechnology Center, National Chung Hsing University, Taichung 402, Taiwan; cjshieh@dragon.nchu.edu.tw; 5 Department of Chemical Engineering, National Chung Hsing University, Taichung 402, Taiwan; ycliu@dragon.nchu.edu.tw; 6 Center for Aquatic Products Inspection Service, National Kaohsiung University of Science and Technology, Kaohsiung 811, Taiwan

**Keywords:** bioactive compounds, *Psidium guajava*, guava leaf extract, biological activity, technological applications

## Abstract

The characteristics of bioactive compounds from guava (*Psidium guajava* L.) leaf extract, their biological activity, and their technological applications are critical topics in many engineering fields. Guava leaf extract is rich in bioactive compounds, including phenolic acids, flavonoids, tannins, terpenes, alkaloids, etc. Bioactive compounds from guava leaf exhibit notable synergistic effects in enzyme inhibition, as well as antimicrobial and anti-inflammatory activities. Natural bioactive compounds are complicated due to their sensitivity and instability during storage, but their use is promising. Thus, for bioactive compound protection, advanced techniques such as the encapsulation, microemulsion, and nanosuspension of such natural bioactive compounds can be a promising approach. These methods are particularly important for the development of natural preservatives serving as additive agents, which have significant industrial relevance. However, sufficient scientific evidence is required to make a health claim on and to promote the functional benefits of guava leaf extract. This review focuses on recent research into guava leaf extract and its technical roles. Demonstrations of the chemical structure of bioactive compounds are addressed, besides discussing their analytical methods, nutritional bioavailability, biological activity, and synergy effects. Furthermore, this review study considers the methods used to protect the active compounds and technological applications in food, pharmaceuticals, and cosmetic products.

## 1. Introduction

*Psidium guajava*, scientifically known as *Psidium guajava* L., is a fruit-bearing tree belonging to the myrtle family (*Myrtaceae*). This species probably originated in Central America and western South America, from Mexico to Argentina [1]. *Psidium guajava* was first introduced to China and other countries such as India, Pakistan, South Africa, Thailand, and Indonesia in the late 17th century [2,3,4]. Presently, *Psidium guajava* is widely cultivated in tropical and subtropical areas and has become naturalized in many countries and regions, such as Southeast Asia, Australia, Central and Southern Africa, and eastern South America [5,6,7]. According to a report by the Food and Agriculture Organization (FAO), the global annual production of *Psidium guajava* was estimated to reach 2.3 million tons in 2023 [3]. The total production of *Psidium guajavas* produced in India is the highest, followed by China, Mexico, Egypt, and Brazil [8].

Globally, there are more than 150 species of the genus *Psidium* as of now [9]. The *Psidium guajava* tree is a woody plant, reaching heights of up to 12 m, including roots, bark, flowers, fruit, and leaves [10]. Among edible fruits, *Psidium guajava* fruit is chosen as a priority by customers because of its richness in vitamin C, taste, and pleasant flavor. Based on the physiology of the ripening process, fruits are classified into two classes: climacteric and non-climacteric. *Psidium guajava,* classified as a climacteric type [11], is unsuitable for prolonged storage and export as a fresh fruit; thus, it is widely used in the production of processed foods such as jam and beverages [12,13]. Moreover, the leaves of *Psidium guajava* are known for their potential in traditional medicine in the form of therapeutic infusions, high-value-added pharmaceuticals, and functional foods [10,14].

*Psidium guajava* leaves demonstrate a higher antioxidant potential compared to *Psidium guajava* fruit [15]. The primary antioxidant substances derived from *Psidium guajava* leaves are polyphenols (galliac acid, caffeic acid, ferulic acid, chlorogenic acid, ellagic acid), flavonoids (kaempferol, quercetin, myricetin, catechin, epicatechin, rutin), terpenoids (limonene, β-caryophyllene), anthocyanins (cyanidin-3-O-glucoside), tannins (ellagic acid, procyanidin B2) and vitamin C, which are well-known to be effective in treating various diseases through powerful antioxidant, anti-inflammatory, and anticancer effects [16]. Key secondary antioxidants, such as myricetin, quercetin, catechin, kaempferol, and rutin, act to prevent or delay oxidation and have powerful antibacterial, antihyperglycemic, anticancer, antispasmodic, and antiamoebic properties. Therefore, *Psidium guajava* leaf-derived products are used to treat dysentery, diarrhea, stomach aches, gastroenteritis, indigestion, diabetes, hypertension, inflammation, rheumatism, fever, lung diseases, and ulcers [17,18]. In recent years, researchers have increasingly focused on the study of various secondary metabolites extracted from *Psidium guajava* leaves to understand their mechanisms of action, precisely evaluate their effectiveness, optimize their use, and prevent side effects.

Extraction is a primary technique for recovering bioactive compounds (BACs) from natural plant sources. This technique is characterized as a separating procedure based on differences in solubility [19]. A solvent works to solubilize and isolate a solute from other materials that have lower solubility in the solvent. Two types of extraction procedures are often identified: solid–liquid extraction and liquid–liquid extraction [19]. The guava leaf extract (GLE) technique involves the mass transfer of bioactive components from solid to liquid form. The aim of the extraction procedure is to maximize the yield of bioactive chemicals from the material while maintaining both its functional and structural integrity [20]. To meet this objective, various advanced extraction techniques, namely ultrasound-assisted extraction (UAE), microwave-assisted extraction (MAE), or enzyme-assisted extraction (EAE), were recently developed to enhance the efficacy of recovering BACs in *Psidium guajava* leaves step by step, replacing conventional methods such as Soxhlet extraction, maceration, or hydro-distillation [19]. In addition, to elucidate the mechanisms of action in GLE for optimizing yield, scale-up issues, and the principle of solute integration, studies have built mathematical models to analyze the kinetic profile and thermodynamic properties of the extraction process, construct artificial intelligence for process optimization, and afterward protect the extracted compounds by the encapsulation process. It is not surprising that recent results are greatly attributed to industrial applications through sustainable extraction methods and technological advancements.

**Figure 1:**
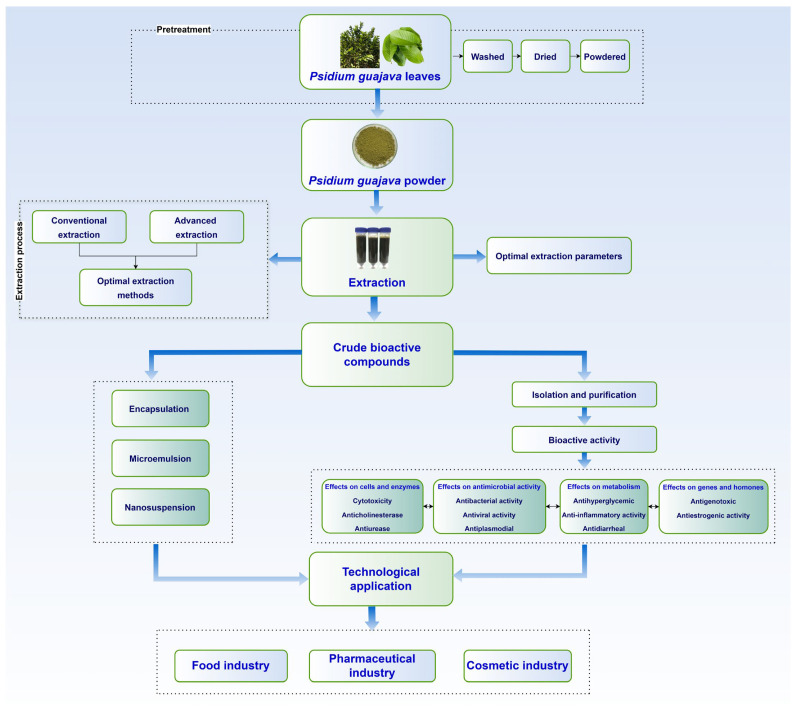
Schematic extraction and technological applications of bioactive compounds from guava leaves (*Psidium guajava* L.).

This review presents a comprehensive systematic study of recent trends in *Psidium guajava* leaf compound extraction and technology applications. The objectives of this article are to address the characteristics of BACs in *Psidium guajava* leaf, their extraction process, and their potential industry applications in functional foods, pharmaceuticals, cosmetics, and for environmental purposes, emphasizing the novelty of the bioactive compounds and of extraction protocols for reproducibility and encapsulation processes (Figure 1).

## 2. Bioactive Compounds in Psidium Guajava Leaf Extract

### 2.1. Nutritional Composition of Psidium Guajava Leaf

Understanding the physical and chemical properties of *Psidium guajava* leaves is an initial step for further biological research. *Psidium guajava* leaves cultivated in Pakistan were determined through the AOAC method by Shabbir et al. [21]. The moisture, fat, ash, fiber, and protein contents were recorded in detail as 82.47% moisture, 3.64% ash, 0.62% fat, 18.53% protein, and 103.05 mg of vitamin C, respectively. These results depend on variables such as the raw material’s source, seasonal and regional differences, and the methodology of extraction [22]. Farag et al. [23,24] highlighted the strong influence of biological, genetic, and environmental variation, year-to-year divergence, and seasonal factors on the chemical components. Therefore, optimizing these factors under controlled conditions is critical for quantifying the key components in *Psidium guajava* leaves [23,24].

### 2.2. Phenolic Compounds

BACs are secondary metabolites derived from the main metabolites of plants, acting as a protective barrier against invading pathogenic microorganisms and demonstrating antioxidant activities, antidiabetic effects, anti-inflammatory actions, cytotoxicity against cancer cells, and antimicrobial activity. These compounds are often found in the roots, bark, and leaves; notably, the total content of BACs in *Psidium guajava* leaves is greater than that in *Psidium guajava* stem bark [25]. Unlike the well-known primary ingredients, BACs are not currently acknowledged or recorded by governmental organizations. To date, over 25,000 BACs have been identified in various sources with diverse chemical structures [26]. These substances may be classified based on their chemical structure, from simple molecules to complex high-molecular-mass polymers, which can be divided into four groups: alkaloids (nitrogen compounds), terpenes, carotenoids, and phenolic compounds [27]. Manikandan et al. [28] used various solvents for BAC phytochemical screening, such as aqueous, ethanol, chloroform, petroleum ether, and hexane extracts. The maximum phytoconstituents were present in the aqueous and ethanolic extracts and exhibited the presence of alkaloids, carbohydrates, tannins, terpenoids, steroidal glycosides, quinones, anthraquinones, saponin glycosides, flavonoids, phenols, total protein, and fixed oil [29]. The difference in the number of phenolic compounds in different solvents might be due to the difference in the chemical nature of the solvent. In the study by Chiari-Andréo et al. [30], the phenolic compounds identified in the 70% ethanol extract of *Psidium guajava* leaves using negative-ion HPLC/MS/MS analysis were analyzed to characterize their phenolic composition (Table 1).

Following that, Xu et al. [23] identified 26 components in BACs with a 2D chemical structure using advanced analytical Ultra-Performance Liquid Chromatography–Quadrupole Time-of-Flight Tandem Mass Spectrometry (UPLC-Q-TOF-MS/MS) in both positive- and negative-ion modes (Table 2). Taken together, the above studies indicated that the phenolic compounds in GLE include subgroups such as phenolic acids, flavonoids, tannins, terpenes, lignans, and coumarins.

#### 2.2.1. Phenolic Acids

Phenolic acids have complex structures consisting of one or more aromatic rings with one or more hydroxyl groups and are divided into hydroxybenzoic acid and hydroxycinnamic acid derivatives [31]. To identify these compounds, structural techniques such as HPLC, GC-MS, and spectrophotometric techniques are widely used with high accuracy and reliability. The Folin–Ciocalteu technique is also commonly used for the quantification of total phenolic content present in GLE because of its simplicity and the ease of interpreting the results, which are expressed as gallic acid equivalents (GAEs) [20,21,22]. The total phenolic content (TPC) commonly analyzed with this technique ranges from 53.24 to 310.98 mg GAE/g (Table 3) [32]. Therefore, *Psidium guajava* leaves are suggested to be a promising natural source of phenolic acids, exhibiting a notably higher TPC compared to *Psidium guajava* pulp and seeds [21].

The primary phenolic acids generally investigated in GLE are gallic acid, chlorogenic acid, ellagic acid, caffeic acid, and ferulic acid. Among these, gallic acid is present in high content. Díaz-de-Cerio et al. [38] determined the presence of specific substances, including gallic acid, in GLE in different states of oxidation (from low to high) using HPLC-DAD-ESI-QTOF-MS techniques. Their analysis reported that the quantified amounts of gallic acid ranged from 153.52 µg/g to 175.90 µg/g of dry leaf weight. Following this, among other phenolics, ellagic acid and chlorogenic acid were determined at medium concentrations. Notably, the concentration of these acids in GLE was lower than that of gallic acid but relatively higher than that of caffeic acid [38,39]. Ferulic acid was identified at average levels in GLE by chromatographic analyses [25].

#### 2.2.2. Flavonoids

The most important phenolic compounds in GLE are flavonoids because of their physiological functions. In fact, the presence of flavonoids is responsible for the development of fragrance, color, and taste in plants. Due to their specific functions, several studies have been conducted on these compounds to evaluate their content, structure, and biological activity. The basic structure of flavonoids has two aromatic rings linked to a heterocyclic ring; afterward, they can be classified into the following groups: flavones, flavanones, flavonols, isoflavonoids, and anthocyanidins. The most common flavonoid types in GLE are flavonols, flavanols, flavanones, and anthocyanins (cyanidin-3-O-glucoside) [38]. Xu et al. [23] identified 16 components of flavonoids in *Psidium guajava* leaves by UPLC-Q-TOF-MS/MS techniques.

In addition, the content of quercetin, kaempferol, guaijaverin, avicularin, and rutin collectively constitutes a high proportion of the total content. A total flavonoid content in the range of 29.66 to 92.38 mg QE/g obtained from GLE was reported in the study by Sam Arul Raj et al. [40]. The results were analyzed with respect to the effect of different *Psidium guajava* cultivars in India (*Allahabad safeda*, *Surka chitti*, *Karela*, and *Lucknow-49*). The study confirmed that the *Karela cultivar* has the highest flavonoid content; the lowest quantity was observed in the ethyl acetate leaf extract of the *Allahabad Safeda* cultivar. Hence, it is important to note that variability in flavonoid content demonstrates significant differences as a function of crop species, growing conditions, and extraction methods [41]. 

#### 2.2.3. Tannins

Tannins are phenolic polymers with a high molecular weight. They are made up of many hydroxyl groups attached to aromatic rings. Tannins can further be divided into different chemical classes, including hydrolysable and condensed tannins (formed from flavan-3-ols monomers). The chemical composition, pharmacological activities, and clinical effects suggest that tannins are a powerful source of bioactivities, especially for antioxidant, antibacterial, enzyme inhibition, and therapeutic applications. To quantify the content of condensed tannins, Farag et al. [42] employed the vanillin method (vanillin–sulfuric acid), in which tannins react with vanillin and sulfuric acid to produce a red-hued complex. The results of the condensed tannins present in GLE were reported at a concentration of 17.79 mg TAE/g dry weight (tannic acid equivalents).

#### 2.2.4. Terpenes and Terpenoids

Terpenes and terpenoids are key components of essential oils derived from plant sources with a significant content, characterized by significant structural and functional diversity. They are widely used as flavoring agents in food and fragrant industries due to their volatility. Indeed, based on these physical characteristics, these compounds enhance the plant’s pollination process and also reduce the risk of harmful organisms. Terpenes are constructed from isoprene units (C_5_H_8_), serving as a fundamental building block. Since terpenes are multiple organic molecules, depending on the number of isoprene units, they could be classified into sub-classes such as monoterpenes (C_10_H_16_), sesquiterpenes (C_15_H_24_), diterpenes (C_20_H_32_), and triterpenes (C_30_H_48_) [31]. Among these, monoterpenes, composed of two isoprene units, represent one of the most common sub-classes of terpenes. Terpenoids, on the other hand, are terpene derivatives formed of several cyclic groups and oxygen that have been modified through oxidation or the combination of hydroxyl or carbonyl groups. These structural alterations result in molecules with diverse biological activities.

Through the GC–MS analysis, Arain et al. elucidated the chemical composition of essential oil extracted from the *Psidium guajava* leaves originating from Pakistan. In this study, a total of 50 components were identified through the hydrodistillation method, while the major constituents of the oils were found to be β-caryophyllene, globulol, nerolidol 2, aromadendrene, cis-α-bisabolene, tetracosane, octadecane, Z,Z,Z-1,5,9,9-tetramethyl-1,4,7-cycloundecatriene, β-bisabolene, limonene, octacosane, δ-cadinene, and 1,4-cadadiene [43]. Notably, when compared to other chemical classes in *Psidium guajava* leaf essential oil, terpenoids were present in a high concentration, accounting for 71.65% of all identified compounds present. The research further revealed that *Psidium guajava* leaves showed a wide range of β-caryophyllene concentrations, which could increase the intracellular accumulation of anticancer agents, thereby potentiating their cytotoxicity due to the absorption of 5-fluorouracil across human skin [43].

These findings about terpenoids are consistent with prior findings regarding their bioactive characteristics. Lima et al. [39] carried out studies to demonstrate the beneficial effect of terpenoids in GLE by investigating the presence of triterpene acids, including betulinic acid, oleanolic acid, and urosolic acid, in *Psidium guajava* leaves. Their emphasis was on the therapeutic potential of these triterpenoids, including anti-inflammatory, anticancer, anti-cholangiocarcinoma, and antitumor activity [25,39,44]. These triterpenoids additionally enhance their antidiabetic activities through mechanisms including aldose reductase inhibition [38].

#### 2.2.5. Alkaloids

Among bioactive compounds in GLE, alkaloids are structurally diverse phytochemical groups. The chemical structure of alkaloids is non-uniform; instead, they are often represented as nitrogen ring structures due to the presence of nitrogen radicals in a heterocyclic ring. Alkaloids can be classified into sub-classes such as pyrrolidine, piperidine, indole, quinoline, and isoquinoline [45]. Due to their diverse chemical structures, they have inspired the development of many modern drugs, such as quinolones, metronidazole, and linezolid. Several investigations have conducted quantitative analyses of bioactive compounds, including sesquiterpenes, saponins, sterols, triterpenoids, phenolics, coumarins, alkaloids, and carotenoids, in *Psidium guajava* leaves [17].

Abd’quadri-Abojukoro et al. [33] reported that *Psidium guajava* leaf contained a high alkaloid concentration compared to twenty-two crude medicinal plant extracts examined, reaching up to 219.06 mg/g dry weight. Alkaloid extraction was conducted by a specific method involving the use of 10% acetic acid in ethanol for extraction, followed by the concentration of the solution. In another study, the alkaloid extraction method was also investigated by changing the type of solvent, for example, ethanol, methanol, and water. Among these, ethanol was noted as an optimal solvent due to its higher efficacy in alkaloid extraction [17,33]. Although some clear results were obtained in the quantitative analysis of alkaloids, there are still many challenges for the future, such as identifying the chemical structure and evaluating the biological properties and synergy effects of these compounds.

## 3. Biological Properties and Synergy Effects

**Figure 2:**
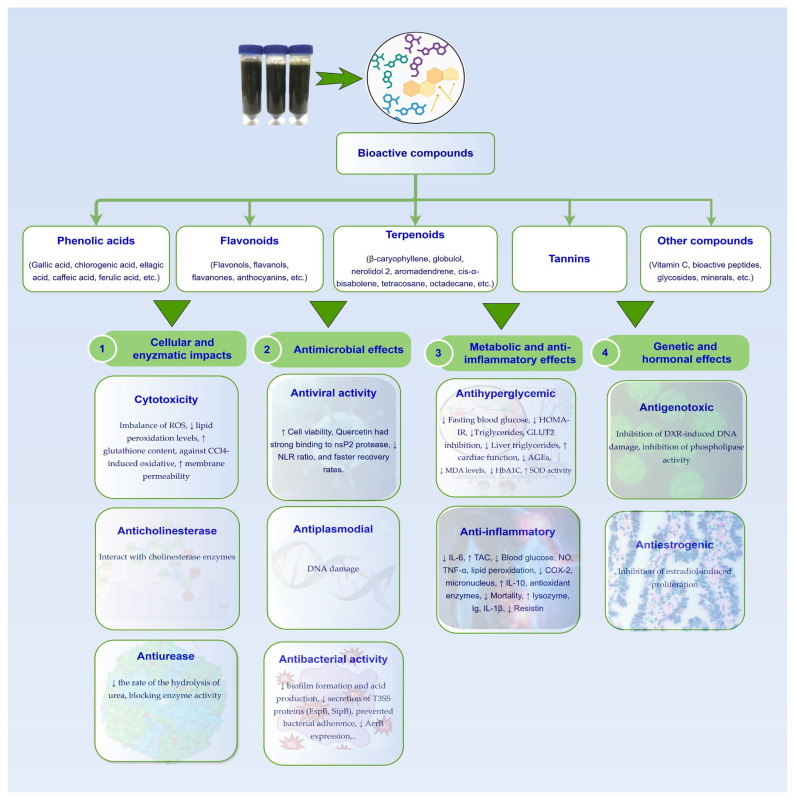
Bioactive activities of Psidium guajava leaf extract.

Scientists have identified the complex composition of GLE by applying phytochemical extraction techniques, as discussed above, and subsequently conducted further research to prove their biological and synergy effects [46]. Interestingly, among the 119 research articles obtained, 44 studies (36.97%) followed this approach, highlighting the diverse biological effects of GLE. According to the experimental models used in these studies, the functions of bioactive compounds in *Psidium guajava* leaf extract are expressed in Table 4. They can generally be divided into four main groups, as shown in Figure 2. The first group is based on cellular and enzymatic impacts, such as cytotoxicity, anticholinesterase, and antiurease activities. The second group is based on antimicrobial effects, namely antibacterial, antiviral, and antiplasmodial activities. The third group further explores metabolic and anti-inflammatory effects, such as antihyperglycemic, anti-inflammatory, and antidiarrheal activities. The last group shows the genetic and hormonal effects, particularly antigenotoxic and antiestrogenic activities. The valuable findings of the collected studies and each biological property of GLE are discussed and presented in this section.

### 3.1. Cytotoxicity

The current literature regarding the toxicity of GLE and its components is based on both in vitro and in vivo studies. Among these, four studies investigated GLE’s in vitro toxicity, focusing on cytoprotective and antioxidant effects. The mechanism of cytotoxicity originates from the uncontrolled multiplication of healthy cells, which is mainly linked to oxidative stress, a state generated by an imbalance of reactive oxygen species (ROS) released during mitochondrial respiration [47,48]. As a result, there is a higher concentration of ROS compared to cellular antioxidants. The flavonoids derived from *Psidium guajava* leaf showed significantly reduced lipid peroxidation levels and enhanced glutathione content, a critical endogenous antioxidant. This pathway mitigates oxidative damage caused by ROS [40]. Isolated quercetin fractions and other components in *Psidium guajava* leaves have been found to inhibit cellular proliferation, have an effect against CCl_4_-induced oxidative, and activate apoptotic pathways. These pathways work by disrupting mitochondrial membrane integrity and increasing membrane permeability, which ultimately leads to programmed cell death [49].

Hepatocellular carcinoma G2 (HepG2) cells are deemed a sensible model for analyzing in vitro xenobiotic metabolism and toxicity to the liver, as they retain most key metabolic functions observed in normal human hepatocytes [40,50]. Furthermore, isolated quercetin fractions, quercetin derivatives (Quercetin-3-O-xylopyranoside and quercetin-3-O-arabinopyranoside) and other components in *Psidium guajava* leaves have been found to inhibit cellular proliferation, have an effect against CCl_4_-induced oxidative stress, and activate apoptotic pathways [51]. These pathways work by disrupting mitochondrial membrane integrity and increasing membrane permeability, ultimately leading to programmed cell death [49].

Given the properties of the compounds in GLE, it demonstrates considerable potential in multiple biomedical applications; especially, it is worth mentioning the potential of *Psidium guajava* extracts in cancer treatment. Under treatment with GLE, biomarkers such as aspartate aminotransferase, alanine aminotransferase, and lactate dehydrogenase levels significantly decreased. This suggests that the extracts may assist in protecting cells from damage [40]. In fact, studies prove that GLE derivatives have potent cytotoxic effects on multiple cancer cell types, including breast, gastric, and colorectal cancers [49]. In another study, Abd’quadri-Abojukoro et al. [52] reported that GLE is a potential plant source of cytotoxic effects. The LC50 value was determined to be 0.0481 mg/mL, with the method of MTT assay (3-(4,5-dimethylthiazol-2-yl)-2,5-diphenyl-tetrazolium bromide) on Vero cells (African green monkey kidney cells), and this value was above the 0.03 mg/mL threshold declared highly toxic set by the U.S. National Cancer Institute. The study further mentioned that GLE has a higher LC50 than other plant extracts, such as Acacia nilotica pod, identified as the most toxic among them (0.0101 mg/mL). The study supports the use of GLE in traditional medical treatment, but unfortunately, it lacks selectivity. In addition, GLE was utilized in the green synthesis of silver nanoparticles, which was carried out in the study by Bar et al. [51]. The authors showed that GLE has significant cytotoxic activity, with an IC50 value of 0.06 mg/mL against MCF-7 (breast cancer) and 0.07 mg/mL against HCT-116 (colon cancer) cell lines.

### 3.2. Anticholinesterase Activity

Flavonoids and phenolic acids derived from the leaves of *Psidium guajava* are among the most important groups of secondary metabolites, as they are considered good sources of natural antioxidants and anticholinesterase activities in human diets [49,53]. Their effectiveness stems from their ability to interact with cholinesterase enzymes, which are potentially associated with Alzheimer’s disease. To explore this potential, the efficiency of GLE against Alzheimer’s disease was investigated by testing their inhibitory effects on two key enzymes: acetylcholinesterase and butyrylcholinesterase. The study employed different solvents, including ethanol, ethyl acetate, and butanol, to extract the active compounds. Among these, ethyl acetate extract demonstrated the highest efficiency in inhibiting acetylcholinesterase (IC₅₀ of 56.11 µg/mL) and butyrylcholinesterase (IC50 of 44.95 µg/mL), as in the case of standard galanthamine. The authors support the use of phenolic compounds due to this significant antioxidant and enzyme-inhibitory activity [53].

### 3.3. Antiurease Activity

Urease is a substantially large nickel-dependent enzyme which is classified under the amidohydrolase and phosphotriesterase groups. In nitrogen metabolism, urease catalyzes the hydrolysis of urea into ammonia and carbon dioxide, thus providing a nitrogen source for microorganisms and plants. Urease is synthesized by numerous bacteria, such as *Proteus*, *Klebsiella pneumoniae*, and *Helicobacter pylori*. In contrast to active urease, enzyme inhibitors, when added to the biochemical reaction, will reduce the rate of the hydrolysis of urea by either blocking enzyme activity by attaching to the active site of the enzyme or interacting with its nickel cofactor, thus changing its structure and function [54]. These agents reduce the production of ammonia and the consequent alkalization of the environment. This disruption of the microbial balance limits the growth of pathogenic bacteria and decreases the risk of complications such as kidney stone formation [55].

Enzyme inhibitors occur naturally in some plants and herbs. However, studies conducted on GLE to demonstrate this activity and consider its use as a source of synthetic antiurease products are still relatively limited to date. The study by Bai et al. [55] reported on the effectiveness of bioactive compounds from GLE with antiurease effects. The results of urease activity inhibition were evaluated among 15 Indian medicinal plants, including *Emblica officinalis* (Indian gooseberry), *Rosa indica* (rose), *Acacia nilotica* (babool), and *Terminalia chebula* (harad), and indicated an inhibition rate of 43.05% for the methanol extract of *Psidium guajava* leaf, with an IC50 of 1.38 mg/mL. In addition, GLE was effective against urease-positive bacteria such as *Pseudomonas aeruginosa* and *Staphylococcus aureus*, resulting in minimum inhibitory concentrations ranging from 125 to 500 mg/mL.

### 3.4. Antibacterial Activity

The antibacterial activity of GLE has been widely studied, focusing on its effects against Gram-negative pathogens such as *Escherichia coli*, *Klebsiella pneumoniae*, *Fusobacterium nucleatum*, *Porphyromonas gingivalis*, *Pseudomonas aeruginosa*, *and Chromobacterium violaceum* and Gram-positive bacteria such as *Staphylococcus aureus*, *Streptococcus mutans*, *Streptococcus gordonii*, *Streptococcus pyogenes*, *Bacillus cereus*, *and Bacillus anthraci* [56]. Key mechanisms include inhibiting biofilm formation, reducing bacterial adhesion, directly affecting bacterial cell membranes, inhibiting acid production, and reducing pH. Regardless of the specific effect on bacterial cell membranes caused by the extract of GLE, Dzotam and Kuete [57] proved that the methanol extract of GLE has antibacterial properties with mechanisms that damage bacterial cell membranes, leading to the efflux of ions, proteins, and other intracellular components, thereby degrading membrane integrity and ultimately leading to bacterial cell death. These authors revealed that the extract possessed exceptional antibacterial properties against both Gram-positive and Gram-negative bacteria, including multidrug-resistant strains. For Gram-positive *Staphylococcus aureus*, the most potent activity was observed (MIC: 62.5 µg/mL). On the contrary, MICs were higher for Gram-negative bacteria such as *Escherichia coli* (MIC: 125 µg/mL) and *Klebsiella pneumoniae* (MIC: 250 µg/mL). In addition, another study showed that the extract also disrupts biofilm formation through decreasing bacterial adhesion on high-density matrices, preventing extracellular polymeric substance synthesis, and lowering acid generation. The extract inhibited acid production by *Streptococcus mutans*, as these bacteria ferment carbohydrates to produce acid, hence decreasing pH and damaging enamel. The MIC for *S. mutans* was documented as 1 mg/mL. The results highlighted the strong antibacterial and antibiofilm efficacy of GLE, especially against Gram-positive bacteria and biofilm-related diseases [58,59].

The ethanol extracts of GLE prevented biofilm formation by *S. mutans* through two key mechanisms, i.e., decreasing bacterial surface hydrophobicity, which reduces adhesion, and inhibiting extracellular polysaccharide (EPS) synthesis, as reported by Phaiboon et al. [60]. At a concentration of 1 mg/mL, the ethanol extract prevented 99.34% of biofilm formation after 24 h (*p* < 0.05), but at lower doses (0.25–0.75 mg/mL), inhibition rates varied from 85% to 95%. Under sucrose-dependent conditions, bacterial attachment decreased to 1.50%, whereas under sucrose-independent conditions, it decreased to 8.43%, both at the same extract concentration of 1 mg/mL [58]. Similarly, the mechanism of biofilm inhibition by *Psidium guajava* extract was found by Gómez et al. [61] for periodontal pathogens, such as *Streptococcus gordonii*, *Fusobacterium nucleatum*, and *Porphyromonas gingivalis*. At a concentration of 1.56 mg/mL, the extract prevented biofilm formation by 77% relative to the control group (*p* < 0.05), demonstrating an effectiveness equivalent to 0.12% of chlorhexidine (positive control). Furthermore, the extract decreased bacterial adherence to host surfaces by 75% (*p* < 0.05). Kenmeni et al. [60] found that methanolic extracts of GLE reduced biofilm development by *Bacillus cereus* and *Bacillus anthracis*. This impact was made easier by the inhibition of EPS production and the disruption of quorum sensing, a vital mechanism controlling biofilm development.

*Psidium guajava* also inhibits the quoruvirulent (QS) system, a key bacterial communication mechanism that regulates virulence factors. Instead of killing bacteria, QS inhibition reduces their pathogenicity by interacting with pigment synthesis. The extract significantly decreased QS-regulated pigments: pyocyanin in *Pseudomonas aeruginosa* by roughly 50% at the lowest concentration when compared to the water extract, prodigiosin in *Serratia marcescens* by 60–70% at the highest concentration, violacein in *Chromobacterium violaceum* by approximately 50% at medium and high concentrations, and staphyloxanthin in *Staphylococcus aureus* by 10–15%. Catechin (50 μg/mL), a recognized QS inhibitor, completely inhibited pigment synthesis in all examined bacteria except for *Streptococcus pyogenes* [62].

The efficacy of bacterial toxin inhibition was described by Nakasone et al. [63], who noticed that GLE did not reduce the synthesis of Shiga toxin by *Enterohemorrhagic Escherichia coli* (EHEC). Their data indicate that the extract lacks a direct effect on the synthesis or release of this toxin as a primary pathogenic factor in EHEC. This information indicates that GLE incompletely reduces bacterial virulence. Notwithstanding this limitation, GLE effectively reduced bacterial virulence [60] and further inhibited the synthesis of virulence proteins linked with the Type III Secretion System (T3SS), an essential system for EHEC to reach host cells and cause illness. The virulence proteins include EspA, EspB/EspD, and Tir. The synthesis of EspB decreased to 10% relative to the control, but the release of SipB and YopB was completely inhibited. By specifically targeting and preventing these proteins, GLE significantly reduces the ability of EHEC to attach to and enter intestinal cells, thereby reducing its overall virulence [63].

It is worth mentioning that GLE inhibits acid production and slows pH reduction in *Streptococcus mutans* cultures by reducing the bacteria’s ability to metabolize sugar, particularly glucose. Based on the glycolytic pH drop assay, the extract decreased the pH drop rate within the first 10 min compared to the control. At the maximum dose (1 mg/mL), the pH decline rate decreased to 0.0453 pH units/min, in contrast to 0.2443 pH units/min observed in the control group. The inhibition of acid production aids in avoiding enamel demineralization, thereby protecting teeth from decay [58,64].

### 3.5. Antiviral Activity

GLE contains bioactive compounds such as flavonoids, quercetin, and antiviral agents, which are therefore proposed to inhibit SARS-CoV-2 replication by targeting RNA-dependent RNA polymerase (RdRp). Molecular docking analysis further revealed that phytochemicals derived from *Psidium guajava* leaves such as Longifollen and quercetin exhibited strong binding affinities with the nsP2 cysteine protease of the chikungunya virus, with minimum binding energies of −8.26 kcal/mol and -6.66 kcal/mol, respectively. GLE increased Vero cell viability by 60% compared to the virus control group, while synthesized silver nanoparticles derived from the extract increased cell viability by 40% [65].

Clinical studies showed significantly higher recovery rates in the GLE group compared to the control group: 49% vs. 27% at 2 weeks and 100% vs. 82% at 4 weeks, with *p*-values of 0.03 and 0.003, respectively [66]. Consequently, the extract suggests its promise as a therapeutic agent for expediting recovery in viral infections. Furthermore, the extract has anti-HIV-1 action, targeting many phases of the virus life cycle. It blocks viral entrance by a mechanism similar to dextran sulfate and reduces HIV-1 protease activity, achieving 74.29% inhibition at a concentration of 0.085 mg/mL. In cell-associated tests, it showed an effective concentration (EC50) of 0.085 mg/mL and a selectivity index (SI) of 21.65, but in cell-free assays, the EC50 was 0.054 mg/mL with an SI of 34.07, demonstrating great effectiveness and low toxicity [67]. These results emphasize the antiviral properties of GLE, indicating its potential as a treatment agent for anti-chikungunya and viral diseases [65].

### 3.6. Antiplasmodial Activity

Natural compounds derived from plants, such as flavonoids, tannins, terpenoids, alkaloids, and saponins, play a great role in combating malaria parasites. Recent studies on extracts from goatweed (*Acanthospermum hispidum*) and Siamese cassia (*Senna siamea*) demonstrate their ability to exert antiplasmodial mechanisms caused by DNA damage in *Plasmodium falciparum* through the comet assay. These agents demonstrated strong antiplasmodial activity, with IC50 values of 3.66 µg/mL (chloroquine-sensitive; 3D7) and 3.70 µg/mL (chloroquine-resistant; Dd2) for *A. hispidum* and 3.95 µg/mL (3D7) and 4.47 µg/mL (Dd2) for *S. siamea*. Likewise, *Alstonia boonei* had significant activity, with IC50 values of 5.13 µg/mL (3D7) and 3.62 µg/mL (Dd2). Conversely, GLE, which includes components such as quercetin and tannins, exhibited diminished antiplasmodial action and did not attain a suitably low IC50 value [68]. Nonetheless, researchers continue to include it as an acceptable option in the investigation and pharmaceutical development of antiplasmodial agents.

### 3.7. Antihyperglycemic Activity

Given the use of *Psidium guajava* leaves as a remedy for diabetes in traditional medicine, it is no surprise that their antihyperglycemic properties have garnered significant research interest worldwide to date [69]. In fact, the attributes that reduce hyperglycemic function have been demonstrated through many mechanisms. One study showed the stimulation of insulin secretion from pancreatic β-cells and the improvement of their function via signaling pathways such as cAMP-mediated Epac2-RyR and GLP-1. After treatment, the extract (1.25 g/kg) was shown to significantly increase serum insulin levels from 0.319 mg/L to 0.600 mg/L after 28 days of treatment [70]. Especially, the extract inhibits glucose transporters, including SGLT1 and GLUT2, resulting in reduced glucose absorption in the small intestine [71]. Müller et al. [72] found that GLUT2-mediated glucose absorption was inhibited by as much as 74% in sodium-free conditions, and in vivo studies showed a fourfold decrease in postprandial glucose levels in treated mice. In addition to controlling glucose, compounds in *Psidium guajava* leaves enhance glycogen synthase (GS) activity and reduce glycogen phosphorylase (GP), resulting in higher glycogen synthesis and decreased degradation. Tella et al. [73] reported an important rise in liver glycogen levels in diabetic rats, exceeding normal values, together with increased GS activity and suppressed GP activity in the treated groups. In addition, the extract had a great effect in enhancing insulin sensitivity by activating the PI3K/AKT/GLUT4 pathway, leading to efficient glucose translocation into muscle and adipose tissues. A study on rats treated with GLE recorded a significant reduction in the HOMA-IR index (from 21.29 to 9.57) [74].

Organic compounds derived from GLE, such as flavonoids and phenolic compounds, have been found to potentially inhibit carbohydrate-digesting enzymes, particularly α-amylase and α-glucosidase [69,75]. Based on the fundamental desire to evaluate the antidiabetic potential of this extract, Diaz-de-Cerio et al. [38] conducted a docking analysis of these isolated compounds to predict molecular shape similarities using the DIA-DB web server. They found that numerous phenolic compounds from GLE, such as naringenin, catechin, and quercetin, had similarities in structure to commonly used drugs for diabetes; moreover, the extract showed antidiabetic potential through interactions with protein targets such as aldose reductase, dipeptidyl peptidase-4 (DPP-4), and peroxisome proliferator activated receptor gamma (PPARG). A further study recently investigated reducing diabetic symptoms by preventing the synthesis of advanced glycation end-products (AGEs) and also lowering the level of their receptor (RAGE), which is attributed to preserving cardiovascular health. One study reported a significant decrease in glycosylated hemoglobin (HbA1C) from 9.67% to 5.64% in diabetic rats after treatment [76].

In terms of lipid metabolism, treatment with *Psidium guajava* leaf not only resulted in lower triglycerides and LDL cholesterol but also increased HDL cholesterol and improved lipid metabolism in a type 2 diabetes rat model [75,76]. In a study on the conventional use of *Psidium guajava* leaf, following treatment with its aqueous leaf extract, Tella and colleagues [73] observed a decrease in LDL cholesterol value from 1.5 to 0.4 mmol/L and an upward trend in HDL cholesterol from 1.3 to 2.4 mmol/L. Moreover, liver inflammation and fat accumulation were reduced under extract treatment via the modulation of pathways such as AMPK and SREBP-1c, which is consistent with the results of Sharma et al. [74]. In their study, the authors observed a decrease in liver triglyceride levels from 188 mg/dL to 177.33 mg/dL after treatment. Furthermore, with a clear lipid-lowering effect, the extract improved digestive enzyme activity and decreased lipid absorption, resulting in a decrease of 19% in triglycerides in the treated groups [71]. Collectively, these data demonstrate the therapeutic potential of GLE in the treatment of diabetes and its related problems through multiple and complementary mechanisms.

### 3.8. Anti-Inflammatory Activity

In the past decade, more attention has been directed toward the anti-inflammatory effect of GLE. Researchers have demonstrated that GLE exhibits potent anti-inflammatory effects through a variety of mechanisms, notably including the inhibition of pro-inflammatory cytokines such as TNF-α, IL-6, and IL-1β, the suppression of inflammatory enzymes like iNOS and COX-2, the reduction in nitric oxide (NO) production, and the enhancement of anti-inflammatory cytokines such as IL-10 [77,78]. These findings highlight the extract’s potential as a natural therapeutic agent for inflammation-related conditions.

Pro-inflammatory cytokines (TNF-α, IL-6, and IL-1β) are critical mediators of inflammation, notwithstanding the role that anti-inflammatory cytokines (IL-10) play in counterbalancing the inflammatory response [79]. GLE has been shown to modulate these cytokines to achieve an anti-inflammatory effect. According to the study of Jayachandran et al. in a rat model [80], this extract reduced TNF-α levels and IL-6 levels by 42% and 37%, respectively, in the pancreatic tissue of STZ-induced diabetic rats. Similarly, Phromnoi et al. [77] observed a significant decrease in TNF-α (48%), IL-6 (45%), and IL-1β (50%) in RAW 264.70 macrophage cells treated with this extract. In addition, regardless of suppressing pro-inflammatory cytokines, the extract enhances anti-inflammatory cytokine production. Ghaderi et al. [81] demonstrated that GLE increased IL-10 levels while reducing IL-6 levels in rats with oral mucosal inflammation, with IL-6 levels decreasing from 1.5 mmol/L to 0.4 mmol/L.

The enzymes COX-2 and iNOS are responsible for synthesizing inflammatory mediators, including prostaglandins and nitric oxide (NO), which increase inflammation. GLE has shown the ability to inhibit these enzymes, thereby decreasing inflammation. Based on in vivo results, Jayachandran et al. [40] found that GLE reduced COX-2 activity in the pancreatic tissue of diabetic rats, while Kumar et al. [82] reported that the extract significantly decreased COX-2 expression (*p* < 0.01) in the liver tissue of rats subjected to radiation damage. Moreover, GLE lowers nitric oxide generation by decreasing iNOS expression. Phromnoi et al. [77] indicated that the extract decreased NO production by 60% in RAW 264.70 macrophage cells, whereas Jayachandran et al. [40] noted an identical drop in NO levels in pancreatic tissues, which correlated with lower iNOS activity.

Oxidative stress in tissues caused by reactive oxygen species (ROS) mainly drives inflammation and tissue damage. GLE, which is rich in antioxidants, reduces oxidative stress by neutralizing ROS and lowering oxidative damage. Ghaderi et al. [81] indicated that GLE enhanced total antioxidant capacity (TAC) in the serum of rats with oral inflammation, assisting in the neutralization of free radicals. Jayachandran et al. [80] similarly observed that antioxidant compounds, including flavonoids and quercetin in *Psidium guajava* leaves, reduced oxidative damage in pancreatic tissue. In addition to reducing oxidative stress, GLE inhibits ROS-activated inflammatory pathways. Wu and colleagues [79] found that the extract decreased oxidative stress in inflamed cartilage cells, hence reducing the release of the inflammatory markers TNF-α and resistin.

The inflammatory signaling pathways NF-κB and MAPK modulate the expression of pro-inflammatory cytokines and enzymes. GLE has shown the ability to block these pathways, therefore mitigating inflammation. One study reported that GLE suppressed NF-κB activation in RAW 264.7 cells; GLE inhibited MAPK signaling molecules, such as ERK, JNK, and p38, resulting in a reduced production of TNF-α, IL-6, and NO, hence enhancing its anti-inflammatory properties [77]. In a similar vein, Jayachandran et al. [80] documented reduced NF-κB activity in the pancreatic tissue of diabetic rats administered the extract.

Apoptosis, also known as programmed cell death, acts as a critical mechanism for eliminating damaged or abnormally inflamed cells. GLE affects apoptosis-related genes and maintains cellular balance. Phonarknguen et al. [44] showed that betulinic acid, a compound present in *Psidium guajava* leaves, boosted the expression of the pro-apoptotic gene Bax by 50% and reduced the expression of the anti-apoptotic gene Bcl-2 by 40%, thereby facilitating the elimination of abnormal inflammatory cells. In addition, GLE maintains cellular membranes and limits vascular permeability, thus reducing edema and reducing tissue damage linked to inflammation. Researchers demonstrated that GLE alleviated vascular damage and preserved liver tissue structure in rats subjected to radiation, suggesting its beneficial impact on inflamed tissues [82]. This extract has advantages comparable to traditional anti-inflammatory medications, and it is safer and associated with fewer adverse effects. This makes it a powerful natural approach for treating inflammation-related disorders.

### 3.9. Synergy Effects

GLE has been shown to exhibit notably synergistic effects when combined with antibiotics or other compounds, as demonstrated by several studies. According to Chouegouong et al. [14], GLE acts as an adjuvant by enhancing antibiotic penetration and protecting antibiotics from inactivation, resulting in a strong synergistic effect with oxytetracycline (FICI = 0.5). Similarly, Dzotam and Kuete [57] observed that GLE reduces bacterial resistance to ciprofloxacin by inhibiting efflux pumps, not only enhancing membrane permeability but also exerting additional impacts on bacterial DNA. The synergistic effects of GLE were specifically pointed out against *Klebsiella pneumoniae* and *Escherichia coli,* with recorded FICI values of 0.37 and 0.50, respectively.

Building on these findings, subsequent research demonstrated that GLE improves the efficacy of antibiotics, including tetracycline, ampicillin, and chloramphenicol, by reducing efflux pump activity (as measured by a 60% reduction in AcrB gene expression) and expanding inhibition zone diameters in combination treatments compared to antibiotics used alone [83]. In addition, studies have shown that GLE interferes with biofilm structures and enhances doxycycline penetration, resulting in improved biofilm inhibition (75% compared to 40% with doxycycline alone) and eradication (60% compared to 30% with doxycycline alone) in chronic bacterial infections [60].

Moreover, another perspective [84] demonstrated that GLE strongly improves natural immunity and survival rates in pangasius fish infected with *Edwardsiella ictaluri*, with survival rising from 35% in the control group to 85% under 0.5% GLE supplementation and reaching 90% when combined with *Phyllanthus amarus*. Together, these research investigations indicate the extract’s capacity to improve antibiotic activity, reduce resistance mechanisms such as efflux pumps and biofilm formation, and enhance immunological responses, therefore proving it a useful additive in medical and functional food applications.

### 3.10. Other Activities

Extracts from *Psidium guajava* leaves or products containing them are commonly used in traditional medicine to treat chronic non-communicable diseases, digestive disorders, and diarrhea [85,86]. However, their specific effectiveness depends on the dosage and method of use. Indeed, observing the antidiarrheal activity of GLE in rat models, the controlled dosages of butanol GLE significantly reduced diarrhea rates and intestinal propulsion, whereas inaccurate dosages of GLE may lead to adverse effects. The impact of GLE on antiestrogenic activity demonstrated a dose-dependent effect, with higher doses providing stronger protective effects against DNA damage and inhibiting estrogen-dependent cell cancer growth. Filho et al. [85] showed in their study that the recovery of oxalic acid concentration in GLE is affected by modifications in the extraction process, which involves parameters such as duration, temperature, and material-to-solvent ratio. High oxalic acid levels can pose health risks. These results supported the importance of optimizing both dosage and method of use to ensure safety and effectiveness.

The antidiarrheal effects of guava leaves are mainly related to bioactive compounds such as flavonoids, tannins, and quercetin. These chemical compounds exert their effects by means of several principal mechanisms: (1) the inhibition of diarrhea-inducing pathogens such as *E. coli*; (2) modulation of intestinal microflora, marked by an increase in beneficial bacteria such as *Bacteroidetes* and a decrease in harmful bacteria such as *Deferribacteraceae*; and (3) reduction in intestinal spasms, assisted by astringent properties that strengthen the intestinal mucosa and lower excessive motility. Observing the properties of butanol extract from guava leaves in a diarrhea model of Kunming mice, it is not surprising that it demonstrated a great antidiarrheal effect, with the loose stool rate decreasing from 46.33% to 1.17%, the diarrhea rate dropping from 100% to 0%, the diarrhea index reducing from 1.74 to 0.27, and the intestinal propulsion rate decreasing from 84% to 53% (*p* < 0.01) [87]. Furthermore, an herbal mixture that included *Psidium guajava* leaves showed antidiarrheal activity with no acute toxicity in Swiss Webster mice. Mice were given oral dosages of the combination or a control solution, and after 14 days of treatment, their internal organs were checked, with no abnormalities observed. LD50 was determined at >5 g/kg, indicating that the combination is safe for human consumption at the recommended dosage (2–4 capsules per day) [86].

Infusions of *Psidium guajava* leaves (from three cultivars: *Pedro Sato*, *Paluma*, and *Roxa*) markedly reduced DNA damage and genotoxicity induced by DXR in human leukocytes. The proportion of nucleoids in severe damage classes 3 and 4 for infusion-treated samples ranged from 0.08% to 0.75%, similar to the negative control (0.83% and 0% for categories 3 and 4, respectively). Conversely, DXR alone caused significant DNA fragmentation, resulting in the classification of 53.67% and 22.84% of nucleoids in classes 3 and 4, respectively [88]. These findings showcase the preventive role of *Psidium guajava* leaf infusions in alleviating DXR-induced genotoxicity. In addition, the protective effects of GLE against genotoxicity have been demonstrated in studies involving caffeine-induced spermatotoxicity and X-ray-induced DNA damage. In caffeine-treated rats, GLE significantly improved sperm quality by reducing sperm head abnormalities from 9.24% to 4.94% at higher doses and increasing sperm count and viability in a dose-dependent manner [89]. In mice exposed to X-rays, pre-treatment with GLE reduced DNA damage, as evidenced by a decrease in micronuclei formation in the proportion of polychromatic erythrocytes from 16.70% to 10.50%. Most importantly, GLE reduced inflammation by lowering COX-2 and IL-6 levels while increasing IL-10, an anti-inflammatory marker [82]. These findings attribute the extract’s strong genoprotective properties to its antioxidant and anti-inflammatory effects.

Guajadial, a chemical derived from *Psidium guajava* leaves, with a structure similar to tamoxifen, has antiestrogenic effects as a selective estrogen receptor modulator. Guajadial was evaluated in vitro on the estrogen-dependent breast cancer cell line MCF-7 BUS utilizing the E-screen assay. In in vivo tests, the results of a uterotrophic assay conducted on prepubescent rats revealed its significant inhibition of estradiol-induced uterine proliferation at doses of 12.5, 25, and 50 mg/kg (*p* < 0.001), without impacting ovarian weight, indicating its specificity for estrogen receptors. Moreover, guajadial prompted cell cycle arrest in the G1 phase, shown by a higher proportion of cells in G1 and a corresponding decrease in the S and G2/M phases at dosages of 2.5 µg/mL and 5.0 µg/mL. Guajadial significantly reduces the proliferative effects of estrogen on estrogen-dependent cancer cells by reducing estradiol’s binding to estrogen receptors and blocking cell cycle progression, highlighting its promise as an effective therapy for these cancers [90]. There are many green solvents used to extract GLE, such as water, ethyl acetate, and ethanol. Some researchers verified the employability of the bioactive activities of *Psidium guajava* leaf extract based on experimental data, and a summary of the mechanisms involved is included in Table 4.

**Table 4:** Summary of bioactive activities and extraction methodologies of Psidium guajava leaf extract.

No	Country	Biological Activity	Solvent Extraction	Research Methodology	Targeted Pathogens	Remarks on Antimicrobial Activities	Reference
**Cytotoxicity**							
1	Brazil	High cytotoxic activity against cancer cells	Essential oil	Cytotoxicity assay	Melanoma (SKMEL-19) HCT116 (colon cancer cells)	IC50: 5.8–12.4 µg/mL	[49]
2	India	Effective antifungal and cytotoxic properties in vitro	Methanol	Cytotoxicity assay	*Bacillus subtilis* *Candida albicans*	MIC: 0.78–1.25 mg/mL	[51]
3	Nigeria	Strong antibacterial activity, particularly against *E. coli*	Ethanol	Microdilution Disk diffusion	*Escherichia coli* *Staphylococcus aureus*	MIC: 1.56–3.12 mg/mL	[52]
**Anticholinesterase activity**							
4	Algeria	Correlation observed between phenolic content vs. antioxidant activity	Ethyl acetate	Antioxidant assays DPPH ABTS CUPRAC		IC50: 4.26 µg/mL	[53]
			n-Butanol			IC50: 5.48 µg/mL	
			Chloroform			IC50 > 200 µg/mL	
**Antiurease activity**							
5	India	Strong urease inhibition (methanol extracts) and potential for treating urinary infections	Methanol Cow urine Aqueous	Urease inhibition assay	*Klebsiella pneumoniae* *Proteus vulgaris*	IC50: 1.25–2.08 mg/mL (methanol is the most effective)	[55]
**Antibacterial activity**							
6	Cameroon	Synergistic effect when combined with antibiotics	Distilled water	Microdilution Checkerboard	*Escherichia coli* *Salmonella enterica* *Clostridium perfringens*	MIC: 1.25–5 mg/mL FICI with oxytetracycline: 0.312–0.5	[14]
7	India	Synergistic effects when combined with antibiotics	Methanol Ethyl acetate	Antioxidant assays Checkerboard	*Salmonella typhi* *Escherichia coli*	MIC: 2–5 mg/mL FICI: 0.5	[40]
8	Cameroon	Focused on multidrug- resistant bacteria	Methanol	MIC/MBC Efflux pump	*Escherichia coli* *Klebsiella pneumoniae* *Pseudomonas ae ruginosa*	MIC: 2–128 μg/mL	[57]
9	Thailand	Effective in preventing dental caries	95% Ethanol	MIC MBC Biofilm	*Streptococcus mutans*	MIC: 1.56 mg/mL no MBC, ↓ biofilm formation vs. acid production	[58]
10	India	Antimicrobial activity against skin pathogens	70% Ethanol	Agar well Diffusion method MIC	*Streptococcus pyogenes* *Proteus species* *Escherichia coli*	MIC ranged from 480.20 to 621.09 µg/mL	[59]
11	Cameroon	Focused on biofilm eradication and reduced antibiotic resistance.	Methanol Ethanol	Biofilm assay MIC	*Bacillus anthracis* *Bacillus cereus*	MIC: 64 μg/mL; strong activity when combined with doxycycline	[60]
12	Peru	Focused on periodontal bacteria	Methanol	Agar diffusion Biofilm	*Streptococcus gordoni* *Fusobacterium nucleatum* *Porphyromonas gingivalis*	MIC: 1.5 mg/mL, significant biofilm ↓ at sub-MIC levels	[61]
13	India	Focused on quorum sensing and virulence reduction	95% Ethanol	MIC MBC	*Pseudomonas aeruginosa* *Staphylococcus aureus* *Serratia marcescens*	MIC: 64–128 μg/mL dependent on extraction method (MAE performed best)	[62]
14	Japan	No impact on bacterial growth	50% Ethanol	ELISA RT-PCR Western blot	*Escherichia coli* *Salmonella enterica* *Yersinia pseudotuberculosis*	↓ Secretion of T3SS proteins (EspB, SipB) and prevented bacterial adherence	[63]
15	Indonesia	Synergism with antibiotics such as tetracycline and ciprofloxacin	70% Ethanol	MIC Efflux pump	*Salmonella typhi*	MIC not specified; ↓ AcrB expression from 11.48 to 7.39 μg/mL	[83]
**Antiviral activity**							
16	India	Anti-chikungunya activity	Aqueous	Vero cell assay Molecular docking	Chikungunya virus (CHIKV)	↑ Cell viability by 60%. Longifollen and quercetin had strong binding to nsP2 protease	[65]
17	Indonesia	Focused on supporting treatment for asymptomatic COVID-19 patients		Clinical	COVID-19 (markers of inflammation in patients)	↓ NLR ratio and faster recovery rates in the *Psidium guajava* extract group	[66]
18	India	Focused on HIV-1 inhibition and ROS scavenging	Methanol	Cell assay EC50	HIV-1 (two different subtypes)	EC50: 0.070–0.085 mg/mL; more effective than Carica papaya	[67]
**Antihyperglycemic activity**							
19	India	Antihyperglycemic, antioxidant	Aqueous	In vivo (rat model)		↓ fasting blood glucose by 32%	[5]
20	Spain	Inhibition of α-amylase and α-glucosidase	Methanol	In vitro		IC50 for α-glucosidase: 0.24–2.6 µM	[38]
21	India	Improved insulin sensitivity	Methanol	In vivo (rat model)		↓ HOMA-IR from 21.29 to 9.57	[65]
22	India	Reduction of lipid absorption	Methanol	In vivo (rat model)		↓ Triglycerides by 19%	[71]
23	Germany	Inhibition of glucose absorption	Ethanol	In vitro In vivo		GLUT2 inhibition up to 74%	[72]
24	Nigeria	Regulation of blood lipids, enhanced glycogen synthesis	Aqueous	In vivo (rat model)		LDL ↓ by 73%, HDL ↑ by 85% ↑ liver glycogen levels by 25%	[73]
25	India	Reduction in non-alcoholic fatty liver disease (NAFLD)	Aqueous	In vivo (rat model)		↓ Liver triglycerides by 5.7%	[74]
26	India	Cardioprotective and antiglycative effects on diabetic myocardium	Ethyl acetate	In vivo (streptozotocin-induced diabetic rats)		↑ Cardiac function and ↓ AGEs	[76]
27	India	Reduction of oxidative stress	Aqueous	In vivo (rat model)		↓ MDA levels from 41.27 to 34.21 nmol/mg	[80]
28	India	Inhibition of advanced glycation end-product (AGE) formation	Aqueous	In vivo (rat model)		↓ HbA1C from 9.67% to 5.64%	[91]
29	China	Activation of antioxidant enzymes	Aqueous	In vivo (rat model)		↑ SOD activity by 20%	[92]
**Anti-inflammatory activity**							
30	Thailand	Anti-inflammatory and antioxidant activities for potential anti-ulcer therapy	70% Ethanol	DPPH, ABTS ELISA for TNF-α, IL-6, IL-1β) LPS-induced RAW 264.7 macrophages		DPPH IC50 = 11.62 µg/mL, ↓ NO, TNF-α, IL-6, IL-1β	[77]
31	China	Anti-inflammatory effects by reducing resistin and TNF-α expression in knee osteoarthritis chondrocytes	70% Ethanol	Articular chondrocytes MTT assay RNA isolation, qPCR for resistin and TNF-α		↓ Resistin (56.59%), TNF-α (51.86%)	[79]
32	China	Anti-inflammatory, antioxidant, and antihyperglycemic effects in diabetic rats	Aqueous	Animal model (STZ-induced diabetic rats) HPLC Enzymatic assays (SOD, CAT, GPx).		↓ Blood glucose, NO, TNF-α, IL-6, lipid peroxidation	[80]
33	Iran	Anti-inflammatory, antioxidant, and wound healing effects on oral mucositis	70% Ethanol	Animal model (rats) Histopathology. ELISA for IL-6 and TAC, DPPH assay	Oral mucositis model	↓ IL-6, ↑ TAC, fibroblast proliferation, thicker epithelium	[81]
34	Vietnam	Immunomodulatory, antioxidant, and disease resistance effects in striped catfish	Ethanol	Fish model Immune assays Proteomics analysis	*Edwardsiella ictaluri*	↓ Mortality (4.76% vs. 47.62%), ↑ lysozyme, Ig, NOS	[84]
35	India	Radioprotective effects, antioxidants, and anti-inflammatory activities against X-ray-induced damage in rats	50% Methanol	Animal model (Wistar rats) Micronucleus assay ELISA for COX-2, IL-6, IL-10 DPPH assay		↓ COX-2, IL-6, micronucleus; ↑ IL-10, antioxidant enzymes	[91]
**Antidiarrheal activity**							
36	Indonesia	Acute toxicity evaluation of antidiarrheal herbal combination		Acute oral toxicity Organ index analysis		No mortality; LD50 > 5 g/kg body weight in mice	[86]
**Antigenotoxic activity**							
37	Brazil	Antigenotoxic, phospholipase and hemolytic activity inhibition	Water (infusion)	Comet assay Enzymatic inhibition tests	Human leukocytes *Bothrops alternatus* *B. moojeni*	75% inhibition of DXR-induced DNA damage; 63.16% inhibition of phospholipase activity	[88]
**Antiestrogenic activity**							
38	Brazil	Antiestrogenic and antiproliferative activity	Dichloromethane	E-screen assay Cell cycle Uterotrophic test	MCF-7 MCF-7 BUS Prepubertal rats	TGI = 2.27 µg/mL (MCF-7 BUS); inhibition of estradiol-induced proliferation	[90]

## 4. Advances in Extraction Technology

Extraction techniques are characterized as separating procedures based on differences in solubility. A solvent works to solubilize and isolate a solute from other materials that have lower solubility in the solvent [19]. The GLE technique involves the mass transfer of bioactive components from solid to liquid form. Two types of extraction techniques are often identified: conventional extraction, such as maceration, decoction, and Soxhlet extraction, and advanced extraction techniques, namely ultrasound-assisted extraction (UAE), microwave-assisted extraction (MAE), vacuum-assisted extraction (VAE), or enzyme-assisted extraction (EAE). The aim of the extraction procedure is to maximize the yield of bioactive chemicals from the material while maintaining both its functional and structural integrity [20]. To meet this objective, various advanced extraction techniques, namely UAE, MAE, VAE, EAE, and alkalinized ethanol extraction, were developed recently to enhance the efficacy of recovering BACs in *Psidium guajava* leaves [93,94].

Maceration is a conventional extraction technique that depends on the naturally occurring diffusion of soluble chemicals from plant materials into a solvent. During this process, the solvent penetrates the plant cell walls and solubilizes the target chemicals, carried by a concentration gradient. Maceration, despite being a simple and low-tech procedure, is time-consuming and less efficient than contemporary techniques. In the general procedure, plant materials are extracted in solvents such as chloroform, ethyl acetate, n-butanol, or ethanol solution at ambient temperature (25 °C) for 24 to 72 h. After finishing the extraction, the solvent is eliminated by a rotary evaporator to concentrate on the extract. Bouchoukh et al. [53] indicated that the extraction yield from maceration ranges from 4.5% to 8.2% for chloroform and ethyl acetate solvents, respectively. On the other hand, Lima et al. [39] determined a 6.1% yield value of triterpene acids when derived by an alkalinized ethanol solvent (2% NaOH in a 95% ethanol solution), calculated based on the dry weight of the leaves. Notwithstanding its limitations, maceration continues to be a common technique because of its simplicity and availability [39].

The decoction technique, a traditional extraction method, is conducted under heat treatment that breaks down plant cells, allowing for the release of bioactive components into the aqueous phase. Water is frequently employed as a solvent because of its efficacy in extracting polar and hydrophilic molecules [95]. This procedure involves boiling 1 g of dried leaves in 16 mL of distilled water until the volume is reduced to 4 mL over a duration of 30 to 60 min. The decoction method, although simple and conventional, yields only a 3.5% extraction efficiency, markedly lower than modern methods such as MAE (performed at 720 W for 140 s using an aqueous solvent), which achieves up to 6.3% [62]. The decreased yield is attributed to the exclusive use of water and high temperatures, which limits the extraction to water-soluble compounds. Lorena and colleagues [95] investigated the key chemical parameters of GLE prepared by the decoction technique. Notably, the decoction extract obtained a high content of TPC (89.58 µgGAE/mg) and TFC (749.42 µgRE/mg), as well as strong antioxidant activity (EC50 7.45 µg/mL). Ten important bioactive components derived from GLE were detected, including catechin, protocatechuic acid, gallic acid, quercetin, epigallocatechin, chlorogenic acid, hyperoside, quercitrin, guaijaverin, and jacoumaric acid. Furthermore, the authors reported that the decoction sample inhibited acetylcholinesterase and 3-hydroxy-3-methyl-glutaryl-CoA reductase with IC50 values of 48.66 and 8.40 µg/mL, respectively. Additionally, the MTT assay demonstrated that neither supernatant exhibited cytotoxicity toward Caco-2 cells. Gallic acid, catechin, chlorogenic acid, hyperoside, and quercitrin permeated a simulated intestinal barrier at rates ranging from 0.21% to 9.95%, whereas other substances permeated it at rates between 0.10% and 0.68%. Taken together, these findings highlight important insights into decoction and its function in traditional therapy.

Soxhlet extraction works through a continuous reflux process, wherein the solvent continuously extracts bioactive components from plant materials. Heat is used in this technique to enhance solubility and diffusion. Consequently, it is well suited for the extraction of thermally stable compounds. A study on Soxhlet extraction carried out continuous extraction for a duration of 48 h using Soxhlet equipment. The results of the extraction yield were found to fluctuate, ranging from 1.80% to 8.07%, with methanol solution being recorded as yielding the greatest results in comparison to aqueous solutions and cow urine. Furthermore, in comparison to other methods such as maceration and decoction, Soxhlet extraction provides a much higher yield, particularly when using organic solvents [55]. However, in terms of time and energy, the method is costly, which may restrict its scalability in certain applications [96].

UAE applies ultrasonic waves to create cavitation bubbles in a liquid, which collapse and create significant shear forces, as it causes cell breakage and then improves the transfer of intracellular compounds into the solvent. UAE is both efficient and time-saving, so it is especially suited for heat-sensitive compounds. To support the use of UAE, Kong and colleagues [97] optimized aqueous ultrasound-assisted extraction conditions for recovering polysaccharides under conditions such as temperature (55 °C), time (30 min), and ultrasonic power (240 W). In these conditions, this procedure attained a yield of 9.2%, significantly outperforming maceration (4.5%) and decoction (3.5%). In addition, a pilot-scale experiment employing a 30 L device with ultrasound power ranging from 300 to 1500 W at a frequency of 20 kHz confirmed the consistency between laboratory and pilot-scale results. These findings demonstrated that UAE is feasible for industrial production due to its high efficiency in polyphenol extraction, as well as its time and energy savings compared to traditional methods. Specifically, large-scale UAE was shown to be a promising method for extracting polyphenols from *Psidium guajava* leaves in industrial applications [98].

MAE is an effective method that uses microwaves to swiftly heat the solvent and plant material, creating intracellular pressure [99,100]. This pressure promotes a breakdown of cell walls, therefore releasing bioactive chemicals into the solvent. Patel et al. [62] conducted the MAE process by determining optimal variables, which included a microwave power of 720 W, an extraction duration of 140 s, and a system of alternate heating and cooling cycles. Based on these optimized parameters, the MAE yield was 12.4%, markedly higher than conventional techniques such as decoction (3.5%) and VAE (8.3%). VAE preserves heat-sensitive compounds, but its yield is not as high as that of MAE. Therefore, MAE, as a fast extraction process, has high efficiency in recovering large quantities of active compounds, primarily attributable to its specific mechanism of rapid heating and effective cell wall breaking.

EAE hydrolyzes plant cell membrane components using enzymes such as cellulose and hemicellulose, thus unlocking phenolic and flavonoid compounds that are normally difficult to extract. This method significantly improves the production of bioactive compounds while preserving an environmentally sustainable and economical strategy for targeting specific molecules. According to Wang et al. [37], EAE employing cellulase, xylanase, and β-glucosidase under optimized conditions (temperature at 50 °C, pH 5.0, extraction time of 12 h, and a solid-to-solvent ratio of 1:4) resulted in remarkable improvements in extraction efficiency. Notwithstanding this, single-use complex enzyme-assisted extraction (CEAE in a 1:1:1 ratio) increased the soluble phenolic content by up to 103.2% compared to untreated samples. Additionally, CEAE elevated quercetin levels by 3.5-fold and kaempferol levels by 2.2-fold. Phenolic extracts from guava leaves obtained via CEAE demonstrated superior DNA-protective effects against oxidative damage induced by Fenton’s reagent, outperforming other extraction methods. The remarkable efficacy of EAE is attributed to its capacity to enzymatically break cell walls, therefore releasing bound phenolic and flavonoid chemicals with increased bioactivity.

In alkaline extractions, ester bonds within plant matrices are hydrolyzed, allowing for phenolic acid dissolution. Following acidification, a precipitation of these compounds occurs, allowing for their effective separation. This method is especially efficient for the selective extraction of certain nonpolar compounds, such as ursolic acid and oleanolic acid. According to Lima et al. [39], the optimized method involves the use of 95% ethanol alkalinized with 2% NaOH as the solvent, with an extraction time of 6 h at room temperature. Following extraction, the pH of the solution is adjusted using HCl to precipitate the triterpene acids. The yields of this extraction achieve 6.10%, primarily focusing on recovering triterpene compounds. However, as is the case with isolating specific compounds, this method proves effective, but it is not general enough to obtain a comprehensive chemical profile from the plant matrix [39].

## 5. Trends and Emerging Technologies

### 5.1. Encapsulation Technology

*Psidium guajava* leaves are classified as a rich source of bioactive compounds. However, these compounds are temperature-sensitive and unstable during processing and storage. Encapsulation techniques can protect these compounds, enhance their stability, and improve their bioavailability [101]. Encapsulation acts by entrapping the substance (core) within a protective barrier (wall materials), with the size of the capsule/core shell ranging from 10 nm to 800 µm [102]. This procedure prevents the degradation, controls the release, and maintains the functional properties of the substance. The advantage of encapsulation is of great importance because of the direct effects of prolonging the shelf life of final products and their wide applicability in the functional food and pharmaceutical industry [50,103].

The advantage of encapsulation is of great importance because of the direct effects of prolonging the shelf life of final products and their wide applicability in the functional food and pharmaceutical industries. Many encapsulation methods have been developed in recent years, including ionic gelation, coacervation, polymer–protein coating, self-feeding, spray-drying, freeze-drying, electro-spinning, electro-spraying, thin-film hydration, ionic gelling, co-extrusion, emulsion system, and solvent removal [101]. These methods can be divided into three classes: physical, physico-chemical, and chemical methods. Among the numerous encapsulation methods available, the most used process for encapsulation is spray-drying due to its ease of scaling up to industry. Some wall material combinations, including maltodextrin and gum arabic, are sprayed into fine droplets within a hot air stream to produce the final product in powder form [104].

Freeze-drying and spray-drying have been widely developed for encapsulating GLE, or *Psidium guajava* leaf oil, within shell materials such as β-cyclodextrin, maltodextrin, and gum arabic [105]. For instance, Jaruporn Rakmai et al. [106] reported that they formed *Psidium guajava* leaf essential oil in a complex consisting of hydroxypropyl-β-cyclodextrin (HPβCD) by freeze-drying it at −50 °C and 1.09 Pa pressure for approximately 48 h. They then analyzed different physicochemical characteristics, such as particle morphology, molecular structure analysis, antioxidant activity, and antibacterial activity, comparing the encapsulated *Psidium guajava* leaf oil with free *Psidium guajava* leaf oil. The derivatization results showed that the use of HPβCD can protect the essential oil. In addition, *Psidium guajava* leaf essential oil encapsulation performed using HPβCD increased its solubility and antioxidant stability by 26–38% when exposed to sunlight and improved its antibacterial activity against *Staphylococcus aureus* and *Escherichia coli* by 4- and 2-fold, respectively.

In encapsulation techniques, the polymer matrix is considered capable of absorbing GLE. The increased nanoparticle size after loading the extract indicates the absorption and adsorption of the extract on the surface of the nanoparticles, such as poly-3-hydroxybutyrate-co-3-hydroxyvalerate, polyurea–formaldehyde microcapsules, and silk fibroin [20]. These encapsulated extracts maintain their antioxidant activity after exposure to high temperatures (70 °C for 24 h), whereas nonencapsulated extracts lose most of their antioxidant properties [103]. These findings are of great interest, as during encapsulation, hydrogen bonding and Van der Waals interactions act as leverage mechanisms. As a result, this reduces the water permeability and swelling of the film, thus enhancing the protection for the active compounds inside [101].

### 5.2. Microemulsion Technology

Microemulsions, or lipid nanoparticles, are novel combinations of water, oil, and amphiphiles that effectively encapsulate essential oils. The combination of surfactants stabilizes a liquid matrix within an aqueous medium, functioning as the basic structure of these lipid nanoparticles. Sutthisawatkul et al. [107] developed microemulsion systems to identify the optimal combination of *Psidium guajava* leaf essential oil, Tween 80 (a surfactant), propylene glycol (a co-surfactant), and water, using pseudo-ternary phase diagrams to determine the optimal formulation. Shelf life analysis revealed that the particle sizes of nanoparticles varied from 10 to 150 nm and remained stable for over 90 days at pH values between 4 and 10. This research demonstrated the stability and efficacy of microemulsions. Furthermore, in comparison to free GLE, microemulsion exhibited higher anti-tyrosinase, antioxidant, and anti-inflammatory properties without causing cytotoxicity to HaCaT cells. The overall results supported the use of microemulsion to improve skin penetration, with water-in-oil systems penetrating more deeply than oil-in-water systems.

The self-nanoemulsifying drug delivery system generally employs mechanisms similar to those of a microemulsion, based on dispersing active compounds in solvents. To address this system’s mechanisms and functionality, Saipriya et al. [108] reported significant findings about the effectiveness of drug delivery systems containing GLE. In their study, the results of the small droplet size identified ranges from 150 to 240 nm. It is most important to increase the interactive surface area of the drug, as well as to enhance its efficient gastrointestinal absorption. This nanoparticle form showed the ability to maximize the pharmacological effects of *Psidium guajava* extract in elevating platelet counts in thrombocytopenic rats. The nanoemulsifying systems also achieved a stable texture without phase separation under pH values from 1.2 to 6.8, thus indicating their suitability for complex digestive conditions. Overall, applying a nanoemulsifying process for GLE has promised advancement in therapeutics, particularly in dengue fever, thrombocytopenia, or immune-related diseases.

### 5.3. Nanosuspension Techniques

Nanosuspension is widely recommended for herbal medicines due to the smaller doses required compared to conventional formulations. Most importantly, the particle surface area and solubility of nanoparticles generally result in an improved bioavailability and enhanced absorbability of bioactive compounds [108]. Nevertheless, research on nanosuspension remains limited. One of the most recent studies is that by Nurdianti et al. [109], who developed a GLE nanosuspension formula. This nanosuspension was constituted using chitosan and sodium tripolyphosphate (as a cross-linking agent) through the ionic gelation method. The characterization results for the optimized formula of *Psidium guajava* leaf nanosuspension at the 0.01% concentration revealed a particle size of 245.70 nm, a polydispersity index of 0.40, and a zeta potential of +26.90 mV. These physical parameters indicated enhanced solubility and bioavailability. Using the agar disk diffusion method, the nanosuspension of 0.01% GLE had an inhibition zone value of 11.45 mm, giving larger inhibition zones than 1% ethanolic GLE of 4.05 mm. The effectiveness of nanosuspensions in terms of antibacterial activity is well recognized, which motivates the pursuit of further advancements in the pharmaceutical industry.

In conclusion, encapsulation is an important technique in the extraction and application of bioactive phenolic compounds in GLE (Table 5). This process helps to protect, control the release, and enhance the efficacy of these compounds in various fields such as cosmetics, food, and medicine.

## 6. Technological Applications

In this section, we summarize the results of other recent publications assessing the application of GLE in various fields, such as foods and beverages, pharmaceuticals, cosmetics, environmental uses, and other applications.

### 6.1. Food Industry

GLE has been recommended as a natural preservative in food processing, offering the best solution to replace artificial preservatives, which may cause harm to human health. To prove its effectiveness in food preservation as a natural preservative, Tran et al. [15] demonstrated this innovative application in preserving frozen striped catfish filets (*Pangasianodon hypophthalmus*) by shelf life tests with three critical indicators for the quality of the filet during storage, such as lipid oxidation, total viable counts, and sensory evaluation. The results indicated that the extract significantly inhibited lipid oxidation, as evidenced by decreased peroxide value (PV) and thiobarbituric acid reactive substances (TBARs). Notably, its flavonoid and phenolic components prevented bacterial growth, decreasing total viable counts (TVCs) during storage, particularly at higher extract concentrations (313 µg/mL). These qualities improved the filets’ prolonged sensory qualities, including color and freshness, for an extended 18-month storage duration at −20 °C. The study reported that GLE is a sustainable, natural alternative to artificial preservatives, which is consistent with the increasing demand for clean-label food preservation solutions.

Another application of GLE is in food packaging, particularly as an edible film-forming polymer [115]. These films or coatings contribute to lowering food waste, cutting plastic pollution, and offering ecological food preservation alternatives without losing quality [12]. In relation to obtaining edible films, Sukoco et al. [115] highlighted the effectiveness of double-layered edible films produced with GLE for the upper layer and a combination of fish oil, zein, and gum arabic for the lower layer. They emphasized the film’s antibacterial activity, antioxidant properties, and physical–mechanical characteristics. The finding indicated the ability of this film to reduce oxidation, inhibit bacterial growth, and preserve food quality during storage. GLE functions to reduce liquid oxidation and peroxide values in the film because they are rich in polyphenol (15.81 mg GAE/g) and flavonoid (6.99 mg QE/g) content. The antibacterial activity of GLE significantly inhibited *Bacillus subtilis* and *Escherichia coli* at a 5% GLE concentration, with inhibitory zones of 40.58 mm and 9.04 mm, respectively. Interestingly, the combination of fish oil and GLE in the film successfully controlled bacterial counts under 3 log CFU/g after 28 storage days. Therefore, the research indicated that GLE may be effectively integrated into the active layer of a double-layer film to enhance its properties while greatly reducing microbial contamination and oxidation rates. This showcases the unique uses of GLE in the development of various food preparation products, including ready-to-eat sausages, cheeses, confectionery, and snack bars.

On the other hand, several authors have reported the use of *Psidium guajava* leaves in functional herbal-based foods. Among them, Jayani et al. [116] conducted a study to formulate functional beverage granules with additives such as stevia, xanthan gum, and maltodextrin and to evaluate these granules by the method of wet granulation. Functional beverage granules derived from a mixture of *Psidium guajava* leaves, purple sweet potatoes, and cinnamon were evaluated based on their physical properties, flow characteristics, and reconstitution behaviors. It is worth mentioning that granules made from *Psidium guajava* leaves and purple sweet potatoes showed good flowability, moisture content, and compressibility, meeting the standard specifications for functional granules (viscosity 100–130 cps, sedimentation volume steady within 30 min, and pH 5.8–6.2). Consequently, *Psidium guajava* leaves may be used in ready-to-drink formulations.

A jelly mixed with GLE was developed to show the extract’s potential application in food products. The study of Sampath Kumar and colleagues [93] examined the proximate, nutritional, and textural properties of jellies using GLE and then compared it to conventional jelly (without the extract). The nutritional composition of the jelly with the extract included 45.78 g of carbohydrates, 3.0 g of protein, 6.15 mg of vitamin C, and 2.90 mg of vitamin B3 and provided a total energy of 120.6 kcal per 100 g serving. These figures were higher than those derived from conventional jelly, reflecting that it could be used as an important energy source in the food industry. The authors also reported on the sensory evaluation of both jellies with respect to the effects of the extract on texture, and it was accepted that the addition of the extract did not affect the end-product texture. Moreover, the enriched jelly again demonstrated antioxidant activity (42.38% for DPPH radicals and 33.45% for hydroxyl radicals) and antibacterial activity, with inhibition zones ranging from 11.40 to 13.60 mm, which aligns with previous study findings. Consequently, the jelly product combined with GLE achieves enhanced nutritional value and provides new applications in functional herbal-based food development and further research.

### 6.2. Pharmaceutical Industry

Despite *Psidium guajava* leaves having a long history of use in traditional medicine, they were only recently applied in the pharmaceutical industry, where studies have demonstrated significant potential. Thombre et al. [117] examined the formulation and efficacy of a cream made with GLE in preventing fungi. Indeed, the authors modified the basic cream formulation, which included beeswax and Tween 80, to evaluate GLE’s impact on stability and drug dispersion efficacy. The cream was then analyzed for attributes such as viscosity, pH, washability, spreadability, antifungal and antibacterial properties, and its potential to irritate the skin of experimental rats. The results highlighted that the hydroalcoholic extract showed the highest flavonoid content and the largest antifungal inhibition zone (27 mm). The cream containing 6% beeswax and 5% Tween 80 exhibited a viscosity of 10,420 cP and a drug diffusion rate of 70%. Its antifungal activity against *Candida albicans*, with an inhibition zone of 25 mm, exceeded that of the standard product (23 mm). As a result, the cream caused no skin irritation and was evaluated as a viable alternative to existing topical antifungal products [118].

The study conducted by Shaheena et al. [94] primarily focused on the extraction of beneficial compounds from *Psidium guajava* leaves for use in dentistry, especially for herbal toothpaste. After the extraction of the compounds, they were used to produce three different types of herbal toothpaste. The authors created three unique formulations, including *Psidium guajava* leaf powder, acacia arabica powder, and fresh coconut oil, among others. Unlike other herbal toothpastes, adding GLE showed significant antibacterial efficacy against oral pathogens such as *Streptococcus mutans* and *Staphylococcus aureus*. In addition, the key product parameters were carefully tested to meet human safety and efficacy parameters, including pH, abrasiveness, and foaming capacity [81]. The research study finally strongly recommended the use of GLE in toothpaste, highlighting its safety, non-carcinogenic properties, and significant medical benefits [64,94].

### 6.3. Cosmetic Industry

A recent innovation in using GLE in cosmetic products is combining the extract with silk fibroin nanoparticles, as demonstrated by Pham et al. [103]. *Psidium guajava* ethanolic extract-loaded silk fibroin nanoparticles protect phenolic compounds, prolong their release times, and enhance their efficacy and usefulness further. When encapsulated in silk fibroin nanoparticles, these compounds exhibited stability even under difficult treatments such as high temperatures (70 °C) for 24 h. In addition, the dual-phase release mechanism of particles facilitated the prolonged use of phenolic compounds for at least 210 min, which is well suited for maintaining their antioxidant abilities in cosmetic formulations. Therefore, this process not only protected the phenolic compounds from degradation reactions but also enhanced their solubility and skin penetration in cosmetic products, prospectively boosting their anti-aging and skin-protective properties as a result.

In the study of Wongsanao et al. [18], evaluating skin care products, the authors investigate the effects of a GLE and menthol toner through a randomized, placebo-controlled clinical trial with 64 participants during thermal regulation post-exercise. Initially, the test toner sample was prepared in the aqueous phase with 20% GLE, 1% menthol, and ingredients such as ethanol and polysorbate 80, while the placebo toner was created using a similar formula but removed GLE and menthol. The key results demonstrated that the toner decreased post-exercise perspiration by approximately 50% compared to placebo values (*p* < 0.05). This was because of the presence of tannins in GLE, which help tighten pores and reduce sweat secretion on the skin’s surface. However, notwithstanding its role in preventing bacteria-related issues, the toner did not negatively impact heat dissipation through the skin, ensuring safety for use. These findings suggested that GLE-based products could be used as an ideal solution for improving hygiene and cooling the body after exercise [18].

Overall, the studies discussed here show that *Psidium guajava* leaf is a source of advantageous compounds with many applications. As a plant-derived antimicrobial agent, it is used in antibacterial creams and oral products within the pharmaceutical industry. In food manufacturing, it serves as an antioxidant and antimicrobial in functional products, beverages, confectionery, or food packaging as a natural preservative. Additionally, it is further used in cosmetics for skin care, or in nanoparticles incorporated to enhance hygiene and sweat reduction. Moreover, its substances provide environmentally sustainable coatings for food preservation and tools for detecting iron in groundwater [70]. These findings not only address the newest and most advanced uses of GLE but also support modern techniques such as nanotechnology or synergistic approaches, which may lead to innovative technological developments in the future.

## 7. Challenges in the Biological Activity of *Psidium guajava* L. Leaf Extract Compounds and Their Technological Applications

Notwithstanding the promising results of GLE, some problems call for further investigation. First, variations in plant origin and development conditions may lead to diverse molecular compositions of GLE, which, in turn, limit the accuracy of its recovery through extraction methods and scaling. Secondly, the absence of established protocols for measuring important bioactive compounds (e.g., quercetin, gallic acid) impacts consistent quality control. In terms of methodology, existing studies have mostly using response surface methodology (RSM) for modeling to optimize extraction parameters. However, as of now, alternative models in machine learning that need to be considered—such as artificial neural networks and convolutional neural networks, which possess the capacity to minimize error variance while improving prediction accuracy—remain poorly examined in this context. In addition, another major gap in current studies is the absence of a cohesive framework that combines theoretical modeling with simulation extraction processes, especially in defining critical variables such as thermodynamic values and mass transfer coefficients. Addressing these factors can reflect the understanding of extraction processes, increase process efficiency, and make it simple to scale it up to industry.

In addition, both microemulsions and nanosuspensions, as advanced encapsulation techniques, require substantial investment and specialist expertise, which may limit their industrial scalability. The thermal and oxidative instability of certain functional phytochemicals during processing and storage reduces their effectiveness in commercial applications. Further, from a regulatory perspective, the approval of GLE-based treatments meets challenges because of insufficient toxicological data and apprehensions about possible allergenicity or combinations with other drugs. Ultimately, the scientific information provided by current investigations remains insufficient to establish a mechanistic insight and nutritional effectiveness. It is also important to emphasize that complex diseases provide big challenges for researchers exploring GLE-based practical applications. Although the role of bioactive compounds and their therapeutic potential has been discussed both in vitro and in animal models, clinical research validating their safety, efficacy, and the optimum dosages of GLE for human use remains limited. Most importantly, recent studies have remained ambiguous in discussing the long-term prospective negative impacts of GLE-based products. By overcoming these barriers, future research could unlock the full potential of bioactive compounds derived from GLE. 

## 8. Future Directions

While the challenges mentioned are considerable, there are great opportunities for further research in the recovery of bioactive compounds. The understanding of the dynamics of the components derived from GLE which offer health benefits remains limited. Therefore, there is an urgent requirement to conduct clinical studies to investigate how they affect human health. In addition, further studies should focus on determining whether their therapeutic effects, which are based on results observed in lab experiments and animal tests can also be replicated in humans, and if so, whether they follow the same biological pathways.

Regardless of the specific extraction process, research in the future should focus on leveraging advanced artificial intelligence (AI) such as machine learning and deep learning as alternative optimization tools for improving the prediction accuracy of the targeted bioactive compound content and for minimizing the error between the predicted and actual output, making the process more reliable. Furthermore, applying mathematical modeling frameworks to demonstrate key parameters, such as kinetic modeling parameters and thermodynamic properties, helps to understand the extraction process nature. In this way, the efficiency and scalability of the method could be enhanced.

With regard to industrial applications, advancements in protective encapsulation systems are important with respect to maintaining the stability and biochemical properties of medicinal plant compounds during processing and storage. It is vital to develop microemulsion and nanosuspension methods which are cost-effective and scalable.

Furthermore, addressing regulatory hurdles through detailed toxicological assessments and allergenicity assessments will be essential for guaranteeing the safety and permitting of GLE-based goods. Ultimately, translating current findings from preclinical investigations to human studies will assist in the development of new approaches and pharmaceutical treatments targeting metabolic disorders and other chronic illnesses. By linking laboratory research with real-life applications, future studies may truly demonstrate the promise of GLE in the food, cosmetic, and pharmaceutical fields.

## 9. Conclusions

This review article is aimed at bringing together new ideas on the plant-derived metabolites of GLE that may have functional and health-enhancing properties with therapeutic potential beyond their basic molecular composition.

This paper discusses the bioactive compounds in *Psidium guajava* L., such as phenolic compounds (gallic acid, chlorogenic acid, ellagic acid, caffeic acid, ferulic acid), flavonoids (quercetin, kaempferol, rutin, catechin, guaijaverin), tannins, terpenes, and terpenoids (β-caryophyllene, limonene, caryophyllene oxide), and alkaloids (piperidine and pyrrolidine derivatives, isoquinoline alkaloids). These compounds are of rising focus because they function widely through cytotoxicity, anticholinesterase, antiurease, antibacterial, antiviral, antiplasmodial, antihyperglycemic, antioxidant, anti-inflammatory, antidiarrheal, antigenotoxic, and antiestrogenic activities. Given their diverse functionality, isolating and recovering these compounds is of high importance, and in this regard, the extraction process of bioactive compounds plays a crucial role. In addition, UAE and MAE are clearly observed as more effective methods than conventional techniques, such as maceration, decoction, or Soxhlet extraction. The common solvents used for extraction are methanol and ethanol, with temperatures between 40 and 60 °C, durations from 20 to 60 min, and material-to-solvent ratios of 1:10 to 1:30 g/mL. Moreover, to preserve the stability of these compounds during extraction, some advanced methods such as freeze-drying, spray-drying, and microencapsulation have been developed with the purpose of improving the stability and nutritional bioavailability of medicinal plant extracts from *Psidium guajava* L. leaf. It is noteworthy that GLE is gaining interest as a natural preservative, particularly for preserving meat and sausages, and as an active ingredient in edible films for extending the shelf life of fruits, vegetables, and processed food products. In food applications, encapsulated GLE with hydrogels and microemulsions has demonstrated effectiveness in protecting the skin, with specific benefits including anti-aging, UV protection, and wound healing. To achieve these effects, studies indicate alginate, hydroxypropyl-beta-cyclodextrin, chitosan, and protein-based compounds as encapsulating materials used.

Collectively, the findings strongly suggest that the chemical profiling of *Psidium guajava* L. leaf-derived metabolites is important in addressing global health challenges, such as combating chronic illnesses, resistance to antimicrobials, and functional benefits in food, cosmetic, and pharmaceutical applications.

## Figures and Tables

**Table 1:** Phenolic compounds in BACs by negative-ion HPLC/MS/MS.

Substance	Chemical Structure	Group	Molecular Formula	Molecular Weight (Da)
3-Sinapoylquinic acid	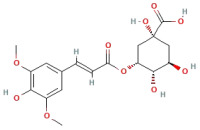	Phenolic acids	C_17_H_20_O_9_	368.11
(-)-Epicatechin 8-*C*-galactoside	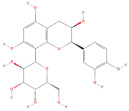	Flavonoids	C_15_H_14_O_7_	306.07
3-Methoxysinensetin	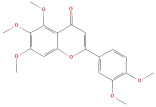	Flavonoids	C_18_H_14_O_8_	358.07
Quercetin 3-*O*-diglucoside and its derivaties	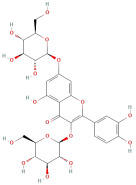	Flavonoids	C_21_H_20_O_12_	464.09
Kaempferol 3-*O*-xylosyl- rutinoside	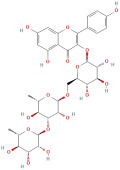	Flavonoids	C_33_H_40_O_20_	756.66
Schottenol ferulate	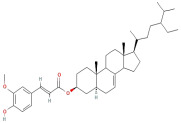	Terpenes	C_39_H_60_O_5_	608.44
Sesamolinol 4′-*O*-β-D-glucosyl (1->6)-*O*-β-D-glucoside	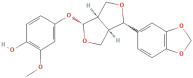	Lignans	C_26_H_36_O_13_	540.56
Esculin	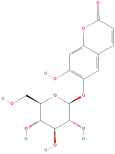	Coumarins	C_15_H_16_O_9_	340.29

**Table 3:** Total phenolic acid content of Psidium guajava leaves from various sources determined using Folin–Ciocalteu method.

Sources	TPC (mg GAE/g)	Extraction Solvent Used	Experimental Conditions	References
Pakistan	83.34	Methanol	Leaves were dried at 50 °C and then extracted in a rotary shaker at 350 rpm for 6 h at 65 °C (methanol), 70 °C (hexane), or 62 °C (chloroform) with a 1:10 (*w*/*v*) solid-to-solvent ratio.	[25]
India	125.77	Methanol	Leaves were extracted with methanol at room temperature, followed by filtration and concentration under reduced pressure.	[32]
Indonesia	79.31	70% Ethanol	Leaves (8 g of dried powder) were extracted with 70% ethanol (1:10 *w*/*v*) via reflux at 70 °C for 30 min, followed by concentration under vacuum.	[33]
Vietnam	145.38	50% Ethanol	Leaves were hot-air-dried at 50 °C for 9 h, extracted with 50% ethanol, and sonicated for 20 min.	[34]
Korea	127.60	50% Ethanol	Leaves were extracted with ethanol (30%, 50%, 70%) for 24 h at 24 °C (1:20 *w*/*v*), followed by filtration and concentration under reduced pressure.	[35]
Thailand	310.98	70% Ethanol	Leaves were extracted with 70% ethanol at room temperature for 4 h, followed by concentration under vacuum.	[36]
China	438.80	Aqueous	Leaves processed through enzyme-assisted extraction using cellulase, xylanase, and β-glucosidase at 50 °C for 12 h, followed by enzyme inactivation at 80 °C for 20 min and drying at 60 °C.	[37]

**Table 2:** Phenolic compounds in BACs detected in both negative- and positive-ion mode.

Substance	Chemical Structure	Group	Molecular Formula	Molecular Weight (Da)
Malic acid	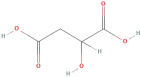	Phenolic acids	C_4_H_6_O_5_	134.09
4-Hydroxybenzoic acid	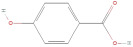	Phenolic acids	C_7_H_6_O_3_	138.12
3,4-Dihydroxybenzoic acid	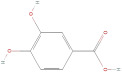	Phenolic acids	C_7_H_6_O_4_	154.12
Coumaric acid	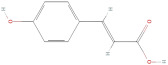	Phenolic acids	C_9_H_8_O_3_	164.16
Gallic acid	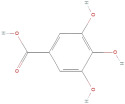	Phenolic acids	C_7_H_5_O_6_	170.12
Caffeic acid	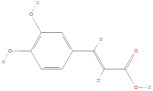	Phenolic acids	C_9_H_8_O_4_	180.16
Ferulic acid	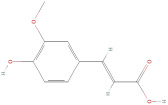	Phenolic acids	C_10_H_10_O_4_	194.19
Chlorogenic acid	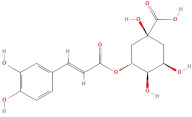	Phenolic acids	C_16_H_18_O_9_	354.31
Formononetin	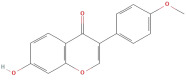	Flavonoids	C_16_H_12_O_4_	268.26
Genistein	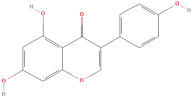	Flavonoids	C_15_H_10_O_5_	270.24
Kaempferol	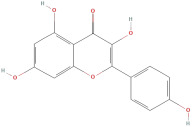	Flavonoids	C_15_H_10_O_6_	286.24
Epicatechin	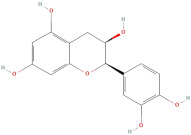	Flavonoids	C_15_H_14_O_6_	290.27
Catechin	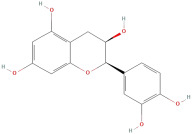	Flavonoids	C_15_H_14_O_6_	290.27
Quercetin	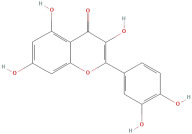	Flavonoids	C_15_H_10_O_7_	302.24
Morin	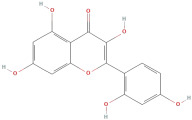	Flavonoids	C_15_H_10_O_7_	302.24
Gallocatechin	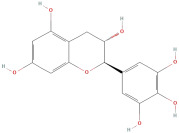	Flavonoids	C_15_H_14_O_7_	306.27
Tamarixetin	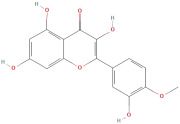	Flavonoids	C_16_H_12_O_7_	316.26
Myricetin	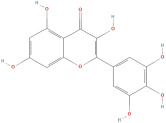	Flavonoids	C_15_H_10_O_8_	318.24
Avicularin	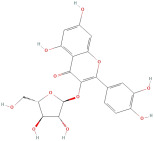	Flavonoids	C_20_H_18_O_11_	434.35
Gossypetin	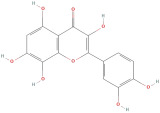	Flavonoids	C_21_H_20_O_13_	464.37
Isoquercitrin	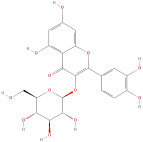	Flavonoids	C_21_H_20_O_12_	464.38
Quercetin 3-O-diglucoside	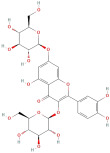	Flavonoids	C_21_H_20_O_12_	464.38
Kaempferol 3-O-glucoside	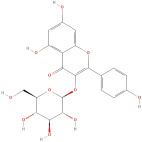	Flavonoids	C_21_H_20_O_11_	448.37
Rutin	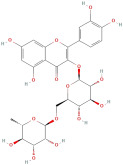	Flavonoids	C_27_H_30_O_16_	610.52
Ellagic acid	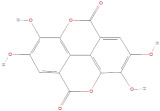	Tannins	C_14_H_8_O_6_	272.20
Procyanidin B2	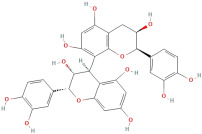	Tannins	C_30_H_26_O_12_	578.53

**Table 5:** Encapsulation of *Psidium guajava* leaf extract.

Method	Wall Materials	Key Findings	Applications	References
Coacervation	Calcium alginate	Produced a multi-functional cotton swab with antibacterial, antioxidant, and UV protection properties.	Textile and biomedical industries	[101]
Polymer–protein coating and self-feeding	Silk fibroin	Preserves the antioxidant activity of GLE and protects the extract from the effects of high temperature.	Food industry and cosmetic industry	[103]
Freeze-drying	Hydroxypropyl-beta-cyclodextrin (HPβCD)	↑ Antioxidant stability by 26–38% when exposed to sunlight and ↑ antibacterial activity against *Staphylococcus aureus* and *Escherichia coli* by 4- and 2-fold, respectively.	Food and cosmetic industries	[106]
Emulsion system	Tween 80, Propylene Glycol	The particle size of the microemulsions ranged from 10 to 80 nm, with enhanced anti-inflammatory activities.	Food and cosmetic industries	[107]
Ionic gelation	Chitosan and sodium tripolyphosphate	The nanosuspension (245.7 nm) inhibited *E. coli* bacteria more effectively than GLE alone, even at low concentrations.	Food industry, pharmaceutical and cosmetic industries	[109]
Thin film hydration	Chitosan, glycerol	Chitosan films containing 2% GLE exhibit antioxidant, antibacterial, mechanical strength, and biodegradable properties.	Food packaging	[110]
In situ polymerization	Poly urea–formaldehyde shell	The treated fabrics exhibited antibacterial activity against *Staphylococcus aureus* but were ineffective against *Escherichia coli*.	Textile and pharmaceutical industries	[111]
Freeze–thaw	Polyvinyl alcohol (PVA) hydrogel	The hydrogel exhibited exudate absorption capacity and antibacterial activity. GLE imparted antibacterial properties to the hydrogel, while PVA is a biocompatible and nontoxic material.	Biomedical industry, particularly in wound care	[112]
Nanoprecipitation	Poly-3-hydroxybutyrate-co-3-hydroxyvalerate	Exhibited antibacterial effects against multidrug-resistant bacterial strains.	Pharmaceutical industry	[113]
Spray drying	Maltodextrin, gum arabic	Maltodextrin mixed with gum arabic was the most effective option for encapsulating the extract.	Food industry	[114]

## Data Availability

No new data were created or analyzed in this study. Data sharing is not applicable to this article.

## Abbreviations

The following abbreviations are used in this manuscript:

ABTS	2,2′-Azino-bis (3-ethylbenzothiazoline-6-sulfonic acid)
AcrB	Acriflavine Resistance Protein B
AGES	Advanced glycation end-products
AI	Artificial intelligence
AKT	Protein Kinase B
CB2	Cannabinoid Receptor 2
CEAE	Cell Extract Antioxidant Enzyme
COX-2	Cyclooxygenase-2
CUPRAC	Cupric Reducing Antioxidant Capacity
DPPH	2,2-Diphenyl-1-picrylhydrazyl
DXR	Doxorubicin
EAE	Experimental Autoimmune Encephalomyelitis
EC50	Half-maximal effective concentration
EHEC	Enterohemorrhagic Escherichia coli
ELISA	Enzyme-Linked Immunosorbent Assay
EPS	Extracellular Polysaccharide
EspB	Escherichia coli Secreted Protein B
FICI	Fractional Inhibitory Concentration Index
GAE	Gallic acid equivalent
GLUT2	Glucose Transporter Type 2
HDL	High-Density Lipoprotein
HOMA-IR	Homeostatic Model Assessment of Insulin Resistance
HRLC-HRMS/MS-QTOF	High-Resolution Liquid Chromatography-High-Resolution Tandem Mass Spectrometry and Quadrupole Time-Of-Flight
IC50	Half-maximal inhibitory concentration
Ig	Immunoglobulin
IL-1β	Interleukin-1 beta
IL-6	Interleukin-6
IL-10	Interleukin-10
LD50	Lethal Dose 50%
LDL	Low-Density Lipoprotein
MAE	Microwave-Assisted Extraction
MBC	Minimum Bactericidal Concentration
MDA	Malondialdehyde
MIC	Minimum inhibitory concentration
MTT	3-(4,5-Dimethylthiazol-2-yl)-2,5-Diphenyltetrazolium Bromide
NF-κB	Nuclear Factor kappa-light-chain-enhancer of activated B cells
NO	Nitric Oxide
NOS	Nitric Oxide Synthase
PCR	Probe-based Polymerase Chain Reaction
PGE2	Prostaglandin E2
PI3K	Phosphoinositide 3-Kinase
PRISMA	Preferred Reporting Items for Systematic Reviews and Meta-Analyses
RNA	Ribonucleic Acid
ROS	Reactive oxygen species
RSM	Response surface methodology
RT-PCR	Reverse-Transcription Polymerase Chain Reaction
SipB	Salmonella Invasion Protein B
SOD	Superoxide Dismutase
STZ	Streptozotocin
Sub-MIC	Sub-minimum inhibitory concentration
T3SS	Type 3 Secretion System
TAC	Total antioxidant capacity
TBARs	Thiobarbituric Acid Reactive Substances
TNF-α	Tumor Necrosis Factor-alpha
TPC	Total phenolic content
UAE	Ultrasound-assisted extraction
UV	Ultraviolet
SipB	Salmonella Invasion Protein B
SOD	Superoxide Dismutase
STZ	Streptozotocin
sub-MIC	Sub-minimum inhibitory concentration
T3SS	Type 3 Secretion System
TAC	Total antioxidant capacity
TBARs	Thiobarbituric Acid Reactive Substances
TNF-α	Tumor Necrosis Factor-alpha
TPC	Total phenolic content
UAE	Ultrasound-assisted extraction
UV	Ultraviolet
